# Retrospectives on Three Decades of Safe Clinical Experience with Allogeneic Dermal Progenitor Fibroblasts: High Versatility in Topical Cytotherapeutic Care

**DOI:** 10.3390/pharmaceutics15010184

**Published:** 2023-01-04

**Authors:** Alexis Laurent, Marina Rey, Corinne Scaletta, Philippe Abdel-Sayed, Murielle Michetti, Marjorie Flahaut, Wassim Raffoul, Anthony de Buys Roessingh, Nathalie Hirt-Burri, Lee Ann Applegate

**Affiliations:** 1 Regenerative Therapy Unit, Lausanne University Hospital, University of Lausanne, CH-1066 Epalinges, Switzerland; 2 Manufacturing Department, TEC-PHARMA SA, CH-1038 Bercher, Switzerland; 3 Lausanne Burn Center, Lausanne University Hospital, University of Lausanne, CH-1011 Lausanne, Switzerland; 4 DLL Bioengineering, Discovery Learning Program, STI School of Engineering, Ecole Polytechnique Fédérale de Lausanne, CH-1015 Lausanne, Switzerland; 5 Plastic, Reconstructive, and Hand Surgery Service, Lausanne University Hospital, University of Lausanne, CH-1011 Lausanne, Switzerland; 6 Children and Adolescent Surgery Service, Lausanne University Hospital, University of Lausanne, CH-1011 Lausanne, Switzerland; 7 Center for Applied Biotechnology and Molecular Medicine, University of Zurich, CH-8057 Zurich, Switzerland; 8 Oxford OSCAR Suzhou Center, Oxford University, Suzhou 215123, China

**Keywords:** biotechnology, burns, cell transplantation program, clinical cell banking, clinical cytotherapies, donor-site wounds, fibroblasts, progenitor biological bandages, regenerative medicine, ulcers

## Abstract

Allogeneic dermal progenitor fibroblasts constitute cytotherapeutic contenders for modern cutaneous regenerative medicine. Based on advancements in the relevant scientific, technical, and regulatory fields, translational developments have slowly yet steadily led to the clinical application of such biologicals and derivatives. To set the appropriate general context, the first aim of this study was to provide a current global overview of approved cell and gene therapy products, with an emphasis on cytotherapies for cutaneous application. Notable advances were shown for North America, Europe, Iran, Japan, and Korea. Then, the second and main aim of this study was to perform a retrospective analysis on the various applications of dermal progenitor fibroblasts and derivatives, as clinically used under the Swiss progenitor cell transplantation program for the past three decades. Therein, the focus was set on the extent and versatility of use of the therapies under consideration, their safety parameters, as well as formulation options for topical application. Quantitative and illustrative data were summarized and reported for over 300 patients treated with various cell-based or cell-derived preparations (e.g., progenitor biological bandages or semi-solid emulsions) in Lausanne since 1992. Overall, this study shows the strong current interest in biological-based approaches to cutaneous regenerative medicine from a global developmental perspective, as well as the consolidated local clinical experience gathered with a specific and safe allogeneic cytotherapeutic approach. Taken together, these current and historical elements may serve as tangible working bases for the further optimization of local and modern translational pathways for the provision of topical cytotherapeutic care.

## 1. Introduction

Topical cytotherapies designed for cutaneous reconstructive surgery or regenerative medicine protocols were among the first to be developed and clinically applied on large scales in the late 20th century. The pioneer works of Reinwald and Green on cultured autologous keratinocytes for the management of burn wounds constitute notable examples [1,2]. During the same time-period, parallel efforts were directed toward the development of various promising cytotherapies for numerous clinical applications. Notable hallmarks of tissue-specific and localized cell therapy administration have been summarized in the works of Brittberg et al. on autologous chondrocyte transplantation, while the stem cell-based Prochymal was among the first approved infusion cytotherapeutic products [3,4]. Historically, high clinical demand and the availability of relatively simple translational models have stimulated academic and industrial efforts toward the eventual clinical use of various topical cell-based and cell-derived therapeutic preparations [5]. Furthermore, two additional dimensions must be considered to best approach the current global ecosystem of cytotherapeutics, namely the recent technical advances in biotechnology and the deployment of numerous modern regulatory constraints [6].

While generally beneficial effects have resulted from the application of recent quality norms (e.g., for manufacturing and control processes) and regulatory requirements in the cell therapy field, several constrictive elements have often limited new technologies’ and applications’ progression from the scientific proof-of-concept state to phase I/II clinical trials [6,7,8,9]. This is best demonstrated by the current relative scarcity in approved cell and gene therapy products, as well as the high rates of retraction for investigative or even approved therapeutic products [6,9]. However, individual consideration of the recently studied and approved cell and gene therapy products indicates that once the clinical safety of a given intervention (e.g., that of Invossa, Kolon TissueGene, Korea) is documented, much regulatory leeway becomes available for the investigators or sponsors [10,11]. Once this important clinical stage is reached, many other aspects of cytotherapeutic product development (e.g., efficacy and efficiency, therapeutic indication adequation) come into play and have historically often caused costly delays, repurposing, or the abandoning of novel cytotherapeutic products altogether [6,12,13,14]. Such negative outcomes can potentially be avoided by gathering specific insights around projects or products under consideration early on in the development phase.

The previously exposed factors and drivers that have shaped the global cell therapy ecosystem have also applied to the clinical use of allogeneic dermal progenitor fibroblasts, which notably constitute cytotherapeutic contenders in Swiss cutaneous regenerative medicine [15,16]. Based on important advancements in the relevant scientific, technical, and regulatory fields, progressive translational developments have slowly yet steadily led to the continuous clinical application of such biologicals and derivatives [5,17,18]. Since 1992, various topical preparations (e.g., semi-solid emulsions, bioactive bandages) containing various forms of progenitor dermal fibroblasts (e.g., proliferation-capable cells, proliferation-arrested gamma-irradiated cells, or cell derivatives) have been successfully clinically used in Lausanne to treat burns, donor-site wounds, and an array of dermatological conditions [18]. Specifically, viable therapeutic allogeneic cells were successfully used for managing adult and pediatric burn patient wounds (i.e., second to third degree burns, skin graft donor site wounds) and refractory lower limb ulcers, with local delivery on resorbable collagen scaffolds (i.e., 9×12 cm in size). Cell-free biological derivatives were also topically used, formulated into oil-in-water emulsions, for the successful management of highly diverse dermatological conditions and cutaneous affections wherein compromised skin structures or abnormal skin reactivity were usually present. While several drastic local adaptations have been necessary to ensure the continued high quality of cytotherapeutic care provision and its regulatory compliance, multifactorial therapeutic gains procured by the considered interventions have enabled the maintenance and development thereof in the clinic [10,16,19].

The overarching goal of this study was to present three decades of acquired clinical hindsight and current perspectives on the use of progenitor dermal fibroblasts and derivatives in Swiss cutaneous regenerative medicine. The retrospective analysis was based on selected internal factors (i.e., versatility and safety of clinical use) and several external factors (i.e., parallels drawn with the current global cytotherapeutic ecosystem) in the interventions under consideration. To set the appropriate context, the first aim of this study was to provide a global overview of approved cell and gene therapy products, with an emphasis on cytotherapies for cutaneous application. Then, to provide a specific point of discussion, the second and main aim of this study was to perform a retrospective analysis on the various applications of dermal progenitor fibroblasts and derivatives, as clinically used under the Swiss progenitor cell transplantation program for three decades. Therein, the focus was set on the extent and versatility of use of the therapies under consideration, their safety parameters, as well as formulation options for simple topical application. Overall, this study firstly demonstrates the strong current interest in biological-based approaches to cutaneous regenerative medicine from a global developmental perspective. Secondly, the subject is discussed more deeply around the example of dermal progenitor cells for clinical use, where important consolidated local clinical experience has been gathered with a safe and versatile allogeneic cytotherapeutic approach.

## 2. Materials and Methods

### 2.1. Data Collection and Overview of the Current Global Cytotherapy Ecosystem

In order to provide an updated and summarized overview of the global cytotherapeutic ecosystem (i.e., currently approved products), multiple sources were cross-referenced. First, several recent scientific literature reviews dealing with regulatory processing and market approvals for cell and gene therapy products were compiled. Then, the relevant national or supra-national online public registries were consulted for the retained products or therapies and for newly approved or retracted products. Finally, the relevant websites of the various product manufacturers or clinical sponsors were reviewed. In cases where conflicting information between available sources was collected, the contents of the official public healthcare agency registries were retained. Data presentation for this part of the study was performed in graphical form, with listing both of the various product classes and of the individual products. The geographical distribution of the various countries of approval for given products and therapies was included as well.

### 2.2. Retrospective Analysis of the Clinical Work on Dermal Progenitor Fibroblasts and Derivatives under the Swiss Progenitor Cell Transplantation Program

The acquisition of previously reported studies (i.e., peer-reviewed scientific and clinical publications) and of the original unpublished data for the present work was performed by the systematic and comprehensive compilation of research, manufacturing, regulatory, and clinical files generated under the Swiss progenitor cell transplantation program since 1991. In particular, the necessary documentary elements were retrieved appropriately from the archives of the Plastic and Reconstructive Surgery Service (CPR) and the Burn Center of the CHUV (Lausanne University Hospital, Lausanne, Switzerland). In detail, the general information, specificities, and data related to cell therapy manufacturing and to related clinical work were mainly synthesized from available regulatory documentation (e.g., local ethical protocols, IMPD, IB, etc.), from manufacturing records, or from treated patient files. With regards to primary patient data, appropriate information anonymization and data codification or security protocols were used at all times during the study.

## 3. Results

### 3.1. The Current Global Cytotherapeutic Ecosystem: Summarized Geographical Distribution of Approved Advanced Therapy Medicinal Products

**Figure 1:**
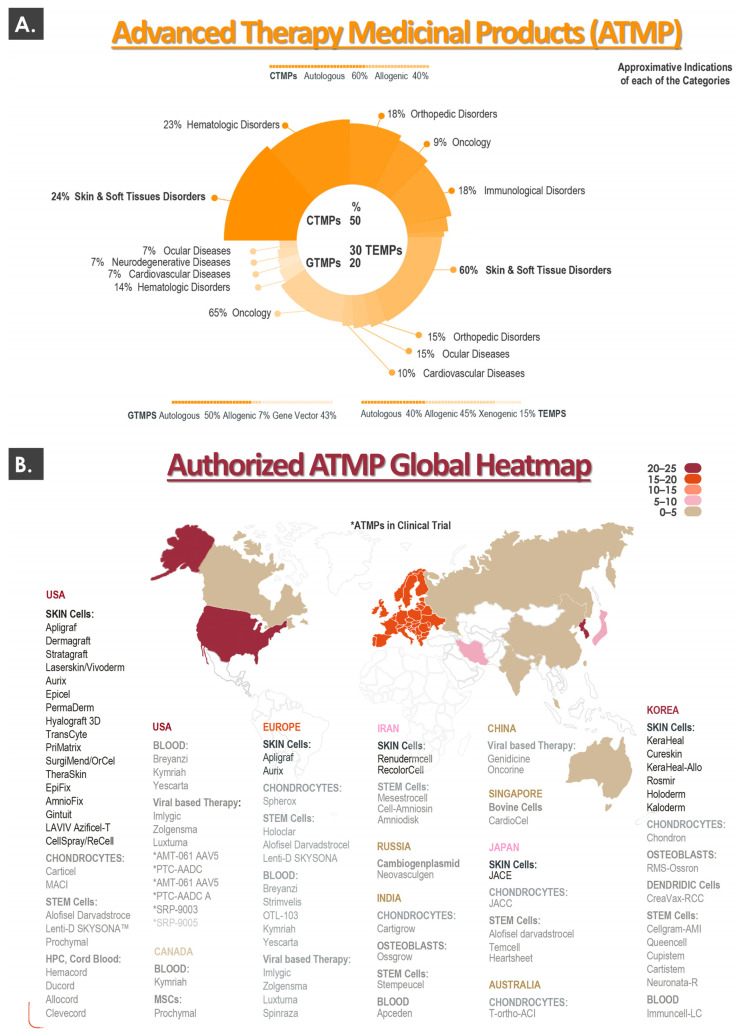
Overview of the current global cytotherapy ecosystem, with a focus on ATMPs for cutaneous application. (**A**) The upper portion of the figure presents a condensed summary of the available ATMPs, distributed into both product sub-categories and therapeutic indications. (**B**) The lower portion of the figure lists most of the authorized ATMPs, along with an illustration of the geographical distribution (i.e., heatmap) of product approvals. ATMP, advanced therapy medicinal product; CTMP, cell therapy medicinal product; GTMP, gene therapy medicinal product; MSC, mesenchymal stem cell; TEMP, tissue-engineered medicinal product.

To set the appropriate general context for the present work, a summarized global overview of currently approved cell and gene therapy products was prepared, with an emphasis on cytotherapies for cutaneous application. A condensed worldwide overview of the currently approved advanced therapy medicinal products (ATMP), distributed into product sub-categories and classified by therapeutic indication, is presented in Figure 1A [6]. A worldwide list of the currently authorized ATMPs, along with an illustration of the geographical distribution (i.e., heatmap) of product approvals, is presented in Figure 1B [6,8,10].

Importantly, a consideration of global ATMP approval statistics reveals that, for cell therapy medicinal products (CTMP), the most important therapeutic indication with regards to product or therapy numbers are “skin and soft tissue disorders” (i.e., 24% of CTMPs, Figure 1A). Similarly, the same indications are specified for 60% of the available tissue-engineered medicinal products (TEMP, Figure 1A). When considering both CTMPs and TEMPs, it may be noted that a large portion (i.e., >40%) of the relevant products is constituted by or comprises allogeneic biological materials (Figure 1A). Analysis of the heatmap describing the global geographical distribution of ATMP approvals reveals that ATMP approval and potential use is mainly and currently restricted to developed countries in the Northern hemisphere (Figure 1B). Furthermore, when considering the relative density of ATMP approvals per country, it appears clearly that most of the recent product developmental efforts have been and are carried out for the North American, European, and South-East Asian markets (Figure 1B).

In addition to this current outlook on approved products and therapies (i.e., ATMPs), the performed cytotherapy ecosystem analysis also took into account some historical elements which predated the ATMP nomenclature and regulatory classification system. Therein, many landmarks and pioneer preparations based on autologous or allogeneic skin cells have been marketed under an array of FDA procedures (e.g., 510(k), PMA, orphan drug, etc.) [20,21]. While some tissue-engineered skin substitutes yielding living cells have been commercially discontinued for a number of reasons, some are still marketed to this day. Notable examples of topical cytotherapeutic preparations harnessing autologous keratinocytes are Epicel, Epidex, Vivoderm, and Myskin [21]. Preparations containing autologous fibroblasts with or without autologous keratinocytes (i.e., and/or other cell types) comprise ReCell, StrataGraft, PermaDerm, LOEX skin, Hyalograft 3D, or MyDerm [21]. Similarly, several preparations containing allogeneic skin cells (e.g., neonatal foreskin fibroblasts and keratinocytes) have been marketed, such as Dermagraft, TransCyte, ICX-SKN, or OrCell (Figure 1B) [21]. In virtually all of the mentioned examples, burn wounds or cutaneous ulcers were listed among the clinical indications [20,21].

In this domain, distinctions are made between acellular and cellular skin substitutes designed for cutaneous wound healing promotion. While acellular products (e.g., Biobrane, Integra, Alloderm) may be effectively used as early and temporary coverage solutions in burn centers, deep and/or extensive burns often require the use of specific cytotherapies [10,22]. The latter may be further subdivided into dermal, epidermal (e.g., stratified keratinocyte sheets in cultured epithelial autografts), and dermo-epidermal (e.g., fibroblasts and keratinocytes in cultured dermal-epidermal autografts) skin substitutes [10]. In the case of burn wounds, the severity and extent of the lesions as well as alternative patient-related factors (e.g., wound anatomic location, availability of skin graft donor sites) dictate the choice of therapeutic skin substitute. Typically, bilayer dermo-epidermal preparations are used in severe burn victims, as multiple cutaneous structures need to be restored, in contrast to epidermal wounds, which may often be managed using stratified keratinocyte sheets [10]. Alternative therapeutic applications of skin substitutes outside of burn centers notably comprise lower extremity ulcers, skin graft donor sites, congenital diseases with cutaneous manifestations, and oral cavity tissue treatments [5,6].

In addition to the developments of novel cell carriers and in vitro biological material processing techniques, it is worth mentioning alternatives to classical split-thickness skin biopsy harvest techniques (e.g., dermatome use), such as those performed in fractional epidermal grafting or epidermal blister grafting [23,24]. Such approaches bear the potential of drastically reducing skin donor site morbidity and enhancing the overall effectiveness of topical cytotherapeutic care for patients presenting burns or ulcers in particular. The use of alternative therapeutic cellular materials (e.g., melanocyte-rich basal cell therapy for vitiligo) may also be considered promising in the domain of clinical topical cytotherapies [24].

Overall consideration of the presented data and specificities on the global cytotherapeutic ecosystem indicates that topical cell-based preparations have always been and continue to be key drivers in recent scientific, technical, regulatory, and commercial developments. Specifically, it was shown that, despite the current existence of well-defined product or therapy classifications and related regulatory guidelines, most of the pioneer registration work in the topical cytotherapy field dates back to the 1990s and early 2000s [21]. Such elements confirm the need for the combined consideration of both current and historical data for a given cytotherapeutic protocol or therapy, as long-term clinical experience and hindsight are critical components of the overall assessment of these interventions.

### 3.2. Global Clinical Work around Dermal Progenitor Cells: International Milestones for the Swiss Progenitor Cell Transplantation Program and Other Clinical Groups

**Figure 2:**
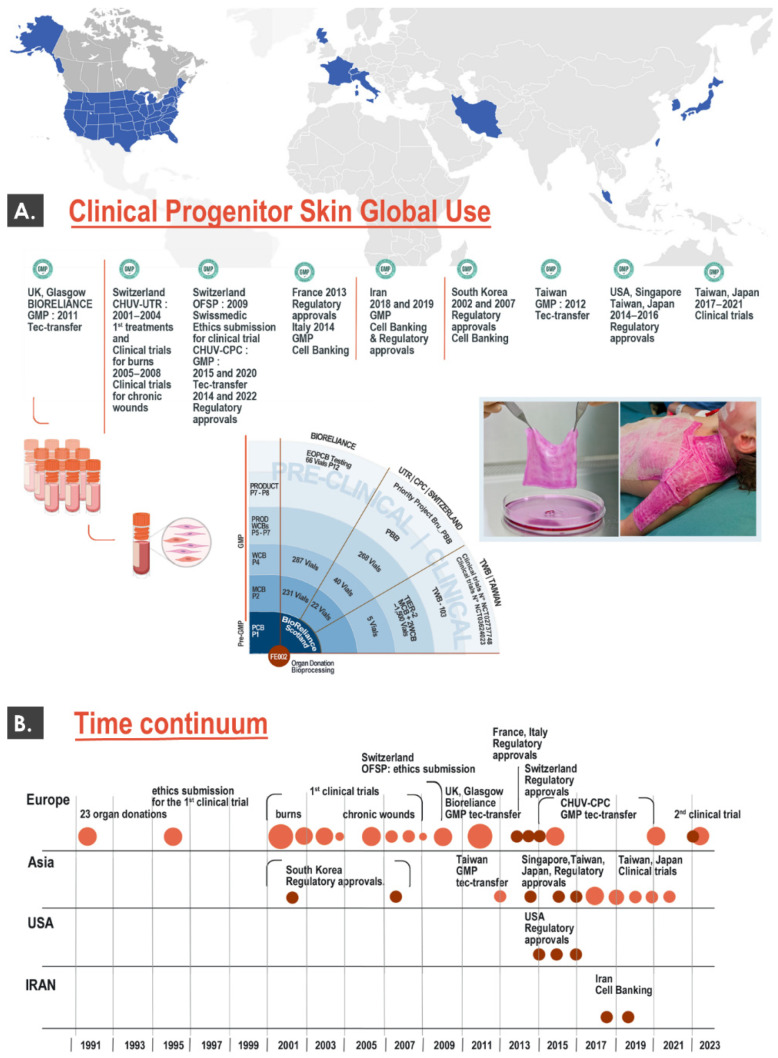
Overview of international developments in the clinical cytotherapeutic use of dermal progenitor cells. (**A**) The upper portion of the figure describes multiple steps of GMP cell banking and regulatory approvals for dermal progenitor cells, under the Swiss progenitor cell transplantation program and in alternative international clinical centers. (**B**) The lower portion of the figure describes major worldwide milestones over the past three decades for the eventual clinical use of dermal progenitor cells, covering the various international examples of clinical work described herein. It is important to note that all mentioned elements not referring to France, Italy, and Iran constitute activities carried out under the Swiss progenitor cell transplantation program. GMP, good manufacturing practices.

As previously mentioned, a restricted number of countries or regions (e.g., USA, Europe, Iran, South-East Asia) have historically been implicated in the development and clinical use of cytotherapies for cutaneous application (Figure 1B). Aside from initial reports on the clinical topical use of dermal progenitor fibroblasts and derivatives in Lausanne dating back to the 1990s, many of the protocols and products currently under investigation have been developed in alternative burn centers (e.g., in Nantes, France or in Tehran, Iran) in the past 20 years (Figure 2) [10,17,18].

Such undertakings were motivated by the high unmet needs of severe burn victims, as well as the high therapeutic potential and technical simplicity of primary progenitor dermal fibroblast cell banking. Specifically, a main component of this converging evolution in therapeutic approaches has been the general homogenization of quality standards for cell therapy manufacture and use, starting with good manufacturing practice (GMP) requirements. Therefore, several groups around the globe have made tangible progress around the use of allogeneic dermal progenitor cells up to the clinical phase for burns and donor site wounds, as summarized hereafter in several relevant randomized clinical trial examples (Table 1).

Firstly, the Tehran University of Medical Sciences (Tehran, Iran) has recently completed a phase I clinical trial (i.e., clinical trial identifier IRCT201302218177N6, 2013–2015) for burn patient donor-site wounds (DSW) using prenatal-derived cellular materials (Table 1). Therein, 10 patients were treated with cultured fibroblasts seeded on acellular amniotic membranes, used as temporary wound coverage solutions [25]. Despite the absence of a significant difference in healing rates between the two membrane groups included (i.e., with and without cells), the safety of the intervention was shown, along with significantly lower recorded pain, fewer infections, and lower inflammation [25]. Secondly, the Nantes University Hospital (Nantes, France) has been conducting a phase I/II clinical trial (i.e., clinical trial identifier NCT03334656, 2018–2023) for DSW using a combination of prenatal-derived cellular materials (i.e., cultured allogeneic fibroblasts and keratinocytes, Table 1) [26]. Therein, 38 patients were treated with fibroblasts and keratinocytes seeded on bovine collagen membranes, used as temporary wound coverage solutions. The aim of this clinical trial is to compare the effects, in the same patient, between a biological dressing (i.e., CICAFAST) and a conventional treatment on DSW healing [26]. The results for this clinical trial are not yet available.

Both of the mentioned clinical trial examples (i.e., by the Iranian and French institutional sponsors) are similar to the clinical work performed under the Swiss progenitor cell transplantation program, albeit with several technical and clinical adaptations [25,26]. Specifically, the selection of the starting biological material, the bioprocessing schemes for cell banking, as well as the formulation approach (i.e., cell type combination, scaffold choice) were assessed as distinct from those reported by the authors of the present study [27,28,29]. It should be noted that no active collaboration exists between the Swiss group and the groups based in France or Iran, and therefore no efforts to actively standardize the procedures have been undertaken to date. Available reports or partial results for the French and Iranian clinical work are comparable to the data gathered in Switzerland, showing the safety of the interventions and positive evolution for selected endpoints [25,26,27,28,29]. From a cytotherapeutic product formulation point of view, such diversity speaks in favor of the previously mentioned versatility of progenitor dermal fibroblasts [18]. This aspect is further substantiated by the use of a hydrogel as a cell carrier in two other clinical trials for DSW and diabetic foot ulcers (i.e., clinical trial identifiers NCT02737748 and NCT03624023, respectively) [5]. These two clinical trials (i.e., 58 enrolled patients overall) constitute important milestones in the international work collaboratively carried out under the Swiss progenitor cell transplantation program, as previously described (Figure 2) [5]. Indeed, an important collaboration exists between the Swiss and Taiwan-based groups, who both currently clinically use the same cell source in their respective clinical investigations (Table 1). Therefore, despite designed differences in formulation options and clinical regimens, it is possible to put forward important efforts toward standardization around the same therapeutic cellular materials. These efforts have been pursued through multiple technology transfers aiming at cell expansion and cell banking standardization, as previously reported [5].

Overall, the fact that multiple clinical groups around the world have been investigating similar dermal progenitor cell sources for a certain number of years (i.e., notably in burn centers) points toward the consolidated safety aspects of the interventions under consideration. Indeed, when reviewing the available information about the clinical work mentioned, no treatment-related adverse outcome or event has to date warranted a study interruption for safety reasons [16,25,26,27,28,29,30,31,32,33,34]. Furthermore, the observed diversity or polyvalence in the existing technical and clinical methodologies has indicated that these safety considerations were applicable to several different cytotherapeutic formulations and clinical situations or settings. Therefore, the existing international body of clinical knowledge around progenitor cell-based topical cytotherapies is currently documented and assessed as highly encouraging from a safety perspective [5,25,26,27,28,29]. Further clinical work is required in all of the implicated centers in order to elucidate the efficacy parameters of the different interventions based on allogeneic dermal progenitor cells. In particular, high importance was identified for outcome definition (e.g., focus on frequency of complications and quality of tissue healing versus rapidity of healing) [16].

### 3.3. Three Decades of Clinical Work around Allogeneic Dermal Progenitor Fibroblasts and Derivatives in Switzerland

At a time when cell therapies were prescribed and administered to patients as magistral preparations in Switzerland (i.e., when harmonized requirements for GMP cell manufacturing and ATMP registration did not yet exist), much leeway was leveraged for the translational development and clinical implementation of novel topical cytotherapeutics [18,30]. Such undertakings (i.e., since 1991 for allogeneic approaches) were based notably on emerging trends in tissue engineering, on local landmarks in cytotherapy, on fundamental biotechnology advancements within the vaccine industry, and were materialized in the form of the Swiss progenitor cell transplantation program [17,18,30]. Registered with local and national health authorities, this translational platform has continuously served as an operational and intellectual property basis for the globalized expansion of primary progenitor cell therapeutic use [18].

In order to provide specific elements on the historical extent and versatility of use for the considered therapies, their safety parameters, as well as formulation options for topical application, summarized data were prepared. Firstly, important methodological elements regarding starting biological material obtention and processing for progenitor cell banking, as well as cytotherapeutic product (e.g., progenitor biological bandages, PBB) manufacture, are presented as supporting information in Figures S1–S4. Similarly, excerpts from preclinical characterization data relative to the dermal progenitor cell sources of interest are presented as supporting information in Figures S5 and S6. Thereafter, the clinical data related to patient treatment using dermal progenitor fibroblasts and derivatives were split into two categories for this study, depending on the topical formulation type and on the nature of the biological materials included. It should be noted that, prior to 2009, several primary progenitor cell types were used therapeutically in the various applications covered herein (i.e., up to four distinct fibroblastic cell types, as described in the individual studies) [5,18,30,31,32,33,34,35]. Then, following transition to GMP bioprocessing and since 2009, all of the clinical work performed under the Swiss progenitor cell transplantation program was performed using the FE002-SK2 cell source, which consists of a fibroblast cell population, manufactured and conserved in a tiered biobanking system [5]. Cell population purity has been shown in published characterization work on the cellular materials of interest, where data on cell surface markers, differentiation potential, and specific protein marker expression (i.e., among other assays) have helped to confirm that the FE002-SK2 cells make up a pure or monomodal cell population [5,28,30].

Firstly, summarized patient statistics (i.e., number of reported patients, in parentheses) for topical treatment using off-the-shelf semi-solid preparations and progenitor cell derivatives were presented hereafter as classified by initial clinical presentation: eczema (5 patients), actopic dermatitis (28 patients), atrophie blanche (3 patients), burns (7 patients), post-skin grafting in burn patients (8 patients), post-skin grafting in ulcer patients (13 patients), scar management (4 patients), severely crevassed and chapped hands (10 patients), radiodermatitis (2 patients), psoriasis (3 patients), rosacea (2 patients), scars and keloids (12 patients), vestibulitis (2 patients), vulvar-vestibulitis (23 patients), vulvar lichen sclerosis (9 patients), atopic dermatitis and dryness (4 patients) [31,32]. Treated conditions also non-exhaustively comprised contact urticaria, contact dermatitis, irritant or allergic contact dermatitis, sunburns or photodermatitis, generalized itch or pruritus, external rectal itch or pruritus, male genital localized itch or pruritus, and localized itch or pruritus due to poison oak or poison ivy exposure and insect bites [31,32]. In almost all of the reported cases, positive evolutions were observed and recorded following topical treatment application [31,32]. Specifically, in addition to the high diversity of the addressed dermatological conditions and the obtention of highly encouraging results in almost all cases, no general or specific treatment-related adverse reaction or events have been recorded [31,32]. Although records and reports exist for >125 patients treated topically with progenitor cell derivatives, it is estimated that several hundred patients have been treated over the years.

Secondly and as concerns the use of viable dermal progenitor fibroblasts (e.g., FE002-SK2 cells) used in combination with collagen scaffolds to constitute progenitor biological bandages, a detailed summary of the cutaneous clinical indications and of the cytotherapeutic product forms used to treat patients under the Swiss progenitor cell transplantation program is presented in Table 2.

Additional details on the various clinical studies performed in Lausanne around the use of the considered progenitor biological bandages are listed in Table S2. Technical details on the preparation and application of the various versions of the considered progenitor biological bandages are presented in Table S3, Figures S7 and S8, and in the Supplementary Document “PBB monograph”. Progenitor biological bandages were developed in Lausanne as an early wound coverage solution to address the temporal and therapeutic gaps existing between initial burn wound stabilization and cutaneous autografting techniques [15,27]. Based on highly encouraging safety and functional results in the Lausanne burn center (i.e., reduced need for skin autografting, improved quality of cutaneous repair), various declinations of pharmaceutical forms and clinical indications have been investigated locally (Table 2) [15,16].

As concerns the therapeutic use of PBB constructs for the management of refractory lower-leg ulcers, the results of a phase I clinical study have shown that patients tolerated multiple treatments, displayed no adverse effects, and ulcers were observed to undergo repair processes similar to those seen in 3rd degree burn victims (Table 2) [34]. Importantly, it was reported that patients with ulcers refractory to compression (i.e., active and passive), hydrocolloids, and skin autografts could be effectively managed to attain wound closure over the course of several weeks or months of follow-up [34]. Such results were considered significant and encouraging, taking into account the type of treated cutaneous ulcer or pathology (e.g., post-thrombotic ulcer, post-thrombotic lipodermatosclerosis, atrophie blanche) and the patient-reported reductions in pain levels [34]. Based on such results, a recent clinical trial was initiated for diabetic foot ulcers (i.e., NCT03624023), wherein the patient follow-up and cutaneous wound healing parameters (e.g., healing rate, time to closure, quality of granulation, wound volume reduction) were specified as outcomes (Table 2) [5].

**Figure 3:**
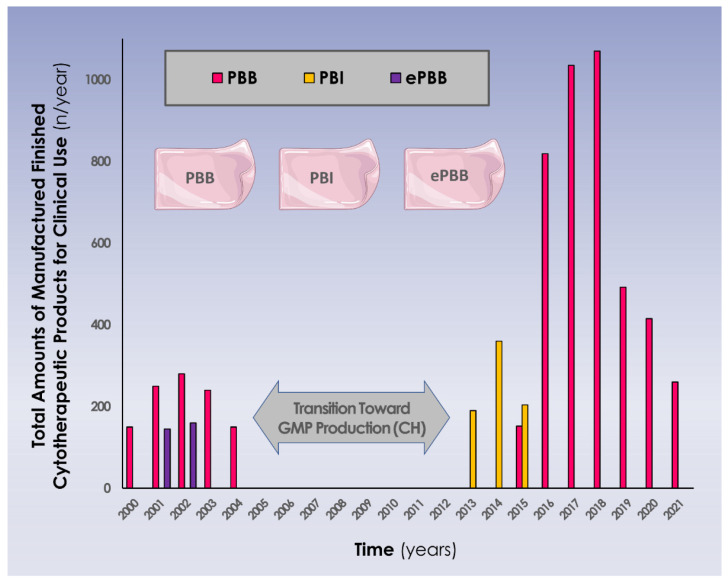
Evolutive overview (i.e., years 2000–2021) of the cytotherapeutic units (i.e., 9 × 12 cm constructs) manufactured for clinical applications in various pharmaceutical forms and in various therapeutic indications. It is to note that years 2020 and 2021 were impacted by the Covid-19 pandemic, with less general activity and fewer accidents occurring in the general population, resulting in reduced numbers of treated patients. Major regulatory shifts and updates occurred between 2005 and 2012, with transition for full GMP manufacturing, which marked a pause in the clinical work in Switzerland, allowing for long-term clinical follow-up work to be performed on the initial patient groups (i.e., 7–10 years of follow-up). CH, helvetic confederation; ePBB, equine progenitor biological bandage; PBI, progenitor biological bandage yielding γ-irradiated cells; PBB, progenitor biological bandage.

An illustrated summary of the evolutive numbers of clinical cytotherapeutic units (i.e., various progenitor biological bandage forms and combined therapeutic indications) manufactured for clinical use in Lausanne under the Swiss progenitor cell transplantation program is presented in Figure 3 (Table S3).

Building on this in-house experience and on successive regulatory approvals (i.e., FDA, TFDA, PMDA, Swissmedic) for further investigational allogeneic cytotherapeutic use, multiple studies and standardized clinical trials have been or are being conducted around the progenitor cells under consideration (Figure 2, Table 2, Table S2). In Lausanne, current practices for progenitor biological bandage preparation and application comprise the extemporaneous reconstitution of cryopreserved dermal progenitor fibroblasts on equine collagen scaffolds. Clinical record excerpts showing the application modalities and wound follow-up for progenitor biological bandages are presented as supporting information in Figures S8–S17. Original data on the number of adult and pediatric patients treated between 2013 and 2021 with PBI or PBB constructs, including data on quantitative exposure to the treatment items, are presented in Table 3.

Overall, the available records show that over 200 patients have been treated with the progenitor biological bandages under consideration for a variety of clinical indications under the Swiss progenitor cell transplantation program (Table 2 and Table 3). Treated patient populations were usually vulnerable or in life-threatening conditions and were extremely diverse, ranging from young pediatric burn patients to geriatric patients presenting refractory lower-limb ulcers [15,34]. Importantly, it was documented that the localized application of progenitor cells often negated the reliance on or need for subsequent skin grafting, acted to limit pain and inflammatory symptoms, and demonstrably reduced the formation of hypertrophic scar tissue (i.e., improved skin biomechanical properties, function, pigmentation balance, as well as gland and follicle functions) [15,16,33].

Overall consideration of the available clinical treatment statistics for dermal progenitor cells and derivatives in Lausanne has outlined the safety and the utility of such highly specialized care provision approaches for >300 patients, all indications and treatment modalities combined. The integration of these three decades of safe clinical experience and the constitution of a wide body of knowledge has enabled us to draw robust conclusions on the retained approach to topical cytotherapeutic care. Parallelly, the historical undertakings summarized herein have contributed to placing primary dermal progenitor cells at the forefront of allogeneic cytotherapies elaborated in Switzerland.

## 4. Discussion

### 4.1. High Versatility of Dermal Progenitor Fibroblasts and Derivatives for Topical Therapeutic Application

Consideration of the data reported herein notably enables an assessment on the high versatility of banked primary progenitor fibroblasts and derivatives for topical therapeutic application in cutaneous regenerative medicine. Versatility was considered firstly from a technical applicability standpoint, as it was demonstrated that both the biological materials and the final product formulations could be easily adapted in view of clinical use. Specifically, both proliferation-capable cells and growth-arrested cells (i.e., lightly gamma-irradiated) could be used in combination with collagen scaffolds to constitute PBBs or PBIs for clinical use, with no reported differences in safety outcomes (Table 3, unpublished results). It should be noted here that the discontinuation of PBIs in favor of PBBs was mostly due to the progressive unavailability of local γ-irradiation capacities, as well as supply chain issues for a specific type of collagen scaffold in 2015. Furthermore, it was also shown that cell-free cellular derivatives could be used in semi-solid topical formulations for clinical use, with no reported adverse events as regards safety outcomes [31,32]. Overall, aggregation of the available data indicated that large margins of technical flexibility exist for biological material processing and for final topical product formulation, without negatively impacting safety parameters and the various product functionalities.

Therapeutic versatility attributes of the considered biological materials have also attested to their robustness and are interesting specifically in the domain of cytotherapeutics, where the definition of a complex product mainly resides in the definition of the ad hoc standardized manufacturing process [5,7]. Therein, low process flexibility, dependence on cold chain maintenance, and product application in clinical settings usually constitute the norm [36]. Therefore, despite several advantages of using fresh cell preparations for acute clinical conditions (e.g., burn patients), numerous advantages characterize a temperature stable semi-solid formulation such as an ointment or cream for outpatient use in chronic dermatological conditions [18,37]. Specifically, the possibility of multiplying product applications and transposing them to a non-clinical setting enables both cost of care rationalization and an augmentation of the long-term quality of care. This last aspect was demonstrated in particular in the case of refractory lower-leg ulcers, where long-term treatment with weekly follow-up is necessary to reliably attain therapeutic resolution [34]. A similar approach may be considered for burn victims, for example, with initial acute treatment using fresh cell preparations, followed by mid-term follow-up treatment using cell derivatives in a simplified topical form [38]. While the treatment modalities of these two phases are different in nature and in objective, the overall goal eventually resides in the obtention of optimally restored structure and function of the impacted cutaneous tissues.

Successful treatment of several veterinary patients using ePBBs as allogeneic cytotherapies has confirmed the technical applicability, safety, and preliminary efficacy of such interventions [35]. This aspect presents tangible potential for future investigative work, as in addition to the global cytotherapeutic ecosystem presented herein for humans, future market interests are predicted to focus on veterinary applications (Figure 1) [35]. Finally, the historically demonstrated versatility aspect of the human clinical work carried out under the Swiss progenitor cell transplantation program may be interpreted as a strength in the current cytotherapeutic ecosystem. Indeed, approved products and therapies may have several yet restricted numbers of indications, addressing specific clinical demands and market niches [9,21]. Therefore, diversified application of dermal progenitor cytotherapeutics as presented herein bears the potential of leading to the tangible rationalization of both regulatory and economic resources.

### 4.2. Extensive/Long-Term Clinical Use of Allogeneic Progenitor Cytotherapies Has Demonstrated Safety and Utility in Complex Cutaneous Wound Care

The integration of all the available data and records relative to the use of primary progenitor cells and derivatives in Switzerland over the past three decades has enabled the sound assessment of their safety, material sustainability, and clinical utility. As presented herein, multiple technical iterations have been performed and several clinical dimensions have been investigated since 1991 (Figure 2, Table 2). As regards the safety aspects of the interventions under consideration, no deaths, clearly identified treatment-related serious adverse events, or specific adverse host reactions were evidenced in preclinical in vivo work or in clinical practice when providing care to hundreds of patients (Table 2) [5]. The safety of the allogeneic application of cutaneous progenitor cells has been further confirmed by collaborating groups and in parallel clinical trials, augmenting the weight of the presented local conclusions [5,25,26].

As regards the efficacy aspects of the progenitor biological bandages administered in Lausanne, the main beneficial effects may be observed in the mid-to-long term after the cytotherapeutic treatment administration [16,33]. Constituting functional yet temporary wound coverages, PBBs may not be assimilated to tissue grafts, as the exogenous cells could not be found in patient tissues following wound healing. However, a 10-year pediatric burn patient follow-up study on the long-term effects of PBBs has demonstrated significantly improved skin viscoelastic properties at the treated wound site [33]. Furthermore, a retrospective case-control study (i.e., comparing PBBs and Aquacel^®^ Ag dressings) has evidenced a trend of reduced needs for corrective interventions or for subsequent skin grafting, as well as significantly reduced hypertrophic scarring (i.e., less scar complications and less corrective interventions) [16]. Generally, PBBs have been documented to reduce the need for skin grafts in the case of large TBSA burns and to generally ameliorate the outcome in the case of complicated (e.g., hand burns) or deep burns (i.e., optimal preparation of the wound bed for grafting and reduction in the infectious risk) [16]. Finally, PBBs have been documented to be effective for the management and resolution promotion of therapy-resistant leg ulcers in geriatric patients (Figures S8–S15) [34].

Overall, given that the considered progenitor biological bandages have been extensively applied (i.e., serially over extended time-periods or in high doses) in various indications where the cutaneous barrier of the patient was destroyed or even absent, an excellent safety profile may be underlined (Table 2 and Table 3). Specifically, the absence of inflammatory and immunological reactions or rejection following the application of allogeneic progenitor cells and derivatives best demonstrates the optimal tolerance thereof. With regard to the long-term therapeutic gains of PBBs, their use as key ancillary treatment modalities within the burn patient care continuum was identified, with a strong focus set on the qualitative aspects of cutaneous tissue healing [16,33].

### 4.3. High Patient Needs and Clinical Demand Remain for Complex Cutaneous Affections: Necessity for Novel and Integrative Biological-Based Therapeutic Solutions

Important clinical needs remain unmet for many cutaneous affections, often underserved due to the difficulty of replacing both skin structure and function or stimulating their repair or regeneration [39,40]. In complex and multi-phasic wound healing, surgical techniques and classical pharmacotherapies often present many limitations with regards to restoration of the cutaneous barrier. Therefore, intense development efforts at the frontiers of surgery, bioengineering, and transplantation science have been deployed over the past half-century, notably in the domain of burn wound care [41,42,43,44,45,46,47]. Novel biomaterial-based or cellular-based therapeutic solutions have demonstrably moved translational and clinical practices several steps toward closing the gap between patient needs and the availability of optimal skin substitutes and wound coverages in cutaneous regenerative medicine [10,19]. Fully bioengineered cutaneous tissue grafts of various designs and applications have been providing new therapeutic solutions and have drastically improved perspectives for DSW and burn wound control, management, and repair stimulation (Figure 1) [6,10].

For the amelioration and simplification of cytotherapeutic product manufacturing workflows, cultured primary progenitor cells derived from cutaneous tissue have been proposed as prime biological starting materials for various forms of standardized cytotherapeutics [5,38]. Specifically, it was demonstrated that an appropriately harnessed allogeneic progenitor cell source (i.e., FE002-SK2 source, derived following a single controlled organ donation) as discussed herein could potentially yield sufficient quantities of safe, standardized, high-quality, and efficient treatment units for the requirements and benefit of millions of patients [5]. As early descendants of stem cells characterized by unipotency, progenitor cells are known to physiologically contribute to tissue homeostasis and repair mechanisms [20,27]. Precise and coordinated biological mechanisms of complex wound healing involving progenitor cells remain incompletely characterized, yet probably rely mainly on multi-factorial paracrine activities (e.g., via the combined actions of low doses of cytokines, growth factors, exosomes, etc.) [37,38,48]. Specifically, FE002-SK2 cells or derivatives were shown to stimulate the proliferation and the migration of primary fibroblasts and keratinocytes in vitro and were reported to contain important proteins and factors implicated in wound healing [38]. Additional suggested mechanisms of action or effects of viable primary progenitor cells used as topical therapeutic agents non-exhaustively comprise the following:

- Intercellular contacts within patient tissues and cells
- Reversal of apoptotic mechanisms and signals resulting from tissular and cellular trauma
- Release of progenitor cell secretomes and related vesicles with signaling functions
- Production and local deposition of extracellular matrix in the wound
- Environment-related specific cellular functions and structural orchestration
- Paracrine modulation (e.g., stimulation of cell proliferation and migration) or trophic action on patient cells and tissues
- Anti-inflammatory and pro-angiogenic effects
- Scavenging of oxidative stress sources

While several of the suggested mechanisms of action of the primary dermal progenitor fibroblasts under consideration have been individually studied in vitro, it is probably a complex effect that is exerted in vivo in patient tissues, enabling the reported pain reduction, cutaneous tissue repair promotion, and prevention of cutaneous scar tissue formation [5,16,18,30].

Overall, the integration of the multiple facets (e.g., historical, scientific, technical, clinical, regulatory, etc.) of cytotherapies for topical use, as well as updated considerations on the relevant product ecosystems, are necessary to ensure an appropriate translation toward the clinic (Figure 1 and Figure 2). In addition, the data reported herein have shown that adaptability and versatility were highly important factors for the maintenance of historical clinical practices, despite multifactorial changes in local and global ecosystems. Therefore, it may be stated that the need for innovation and advancements in the field of cutaneous regenerative medicine should always be addressed in the current context, conjugated with sufficient hindsight, and guided by appropriate retrospective considerations, if possible.

### 4.4. Navigating the Evolving Swiss Regulatory Ecosystem for the Provision of Safe and Standardized Allogeneic Progenitor Cytotherapies for Burns and Wounds

At the time of the initial clinical work set forth under the Swiss progenitor cell transplantation program (i.e., starting in 1991), the relevant regulatory jurisdiction was mainly cantonal (i.e., ethics commissions, cantonal chemists), assorted to centralized program registration with federal health authorities [17]. Later, major and disruptive updates took place in 2007, with the instauration of renewed regulations which trickled down from European practices, as well as the entry into effect of new Swiss laws [19]. These proceedings yielded a direct impact on local clinical work, due to the necessity to adapt material bioprocessing schemes and cytotherapy manufacture to GMP standards (Figure 3, Table 3). Historically administered as magistral preparations under hospital exemptions or compassionate use, PBBs are currently considered as standardized transplant products under Swiss laws or as combined advanced therapy medicinal products (cATMP), entailing specific manufacturing requirements and registration pathways [16,19].

Consideration of the consolidated clinical work around dermal progenitor cells and derivatives presented herein should be performed, while keeping in mind the vast timespan covered by said work (i.e., three decades). Namely, the historical portion of the presented clinical work is not meant to be evaluated from a current regulatory perspective, due to the aforementioned important legal and regulatory shifts. Appropriate legal and regulatory provisions were followed at the time of treatment administration to each patient. Notwithstanding this, the fluidity in the local regulatory frameworks has contributed to shape the developmental approach and the clinical therapeutic work around PBBs, which iteratively evolved for quality optimization and updated regulatory compliance reasons (Figure 3) [5]. This aspect is worth mentioning as the successive updates in specific regulations and requirements have created many bottlenecks (i.e., increased costs, complex procedural processes) over the years, negatively impacting many historical therapeutic practices around the world [6,8,9,10]. Therefore, a high intensity of local multidisciplinary work has been necessary for the maintenance in Lausanne of the reported clinical practices, driven mainly by the high clinical demand for therapeutic solutions in vulnerable patient populations. Specifically, direct impacts of PBB applications on vital outcomes (i.e., rescue of extreme burn patient cases) and patient life quality parameters (i.e., averting the need for DSW creation or enabling multiple autologous skin harvests) were practically documented and continue to justify emergency medical use to this day.

When considering the influence of regulatory bodies on the global cytotherapy ecosystem, many hurdles have been identified in the European or North American regulatory ecosystems pertaining to translational development of biologicals (i.e., especially by academic centers) [7,8,9,10]. Indeed, the new developments in and registration of novel cytotherapies must be the subject of stringent safety and quality standards (i.e., following industry best-practices) to guarantee the provision of high-quality and non-iatrogenic clinical cytotherapeutic care [5,19]. Therefore, dialogues and collaboration with local and national health authorities and regulators appears as a critical component for the continual reshaping of specific regulatory ecosystems. Such approaches are necessary to ensure a continuity and the maintenance of safe and proven cell therapies in clinical practice, for their eventual benefit to patient care.

### 4.5. Technical Limitations and Clinical Hindsight on Topical Progenitor Cytotherapeutics: Pharmaceutical Solutions and Margins of Optimization for Future Work

A consistent technical limitation has been evidenced around the use of extemporaneously reconstituted PBBs, namely the cumbersome and costly cold chain maintenance for cryogenic storage and shipping [16]. Indeed, such practical requirements limit the number of clinical centers potentially using the technology, as specific infrastructure, equipment, and trained personnel are required. Additionally, the product reconstitution process and related logistical constraints generally require that patients are hospitalized or come back to the hospital for repeat treatment and maintenance treatment, technically limiting the number of product applications to a minimum. High importance is therefore set on the dosing regimen of the cytotherapeutic product or therapy, not in terms of absolute dose, but in the correct and repeated application thereof.

A key parameter in ensuring clinical success in topical cytotherapeutic care may reside in the use of multiple small product doses administered regularly over an extended time-period, for production of enhanced results as compared to few large doses. Based on this concept and on preliminary in-house clinical observations, the considered progenitor cell extracts were formulated into semi-solid topical formulations for wound repair promotion [31,32]. Further clinical research and development efforts have historically yielded several generations of Swiss-designed topical preparations for various uses, commercialized under several brands (e.g., https://www.neocutis.com/, accessed on 9 September 2022) over the past decades and attaining global reach [5,18]. Based on such experience gathered by the authors, multiphasic care provision in complex clinical cases is currently considered, with initial cytotherapeutic treatments using PBBs or analogs, followed by functional maintenance treatments. The latter is being developed using technological derivatives and adapted product formulations meeting patient needs and possibilities of ambulatory self-application.

As regards the endpoints and readouts of cutaneous clinical care and patient follow-up, specific parameters have been identified by the authors, for the tangible and optimal assessment of the effects of the cytotherapeutics and derivatives under consideration. As mentioned, appropriate follow-up and maintenance treatments are vital in ensuring appropriate cutaneous wound healing, including the elimination of aggravating factors (i.e., behavioral, professional occupation), if possible. Furthermore, temporal and qualitative healing parameters should be defined with great care, as clinical investigators should focus specifically on the overall skin quality and functionality following repair and on patient quality of life following the cytotherapeutic interventions [16]. Overall, such parameters may prove to be more important for the reduction of global socio-economic burdens than classically employed primary endpoints, such as time to initial wound closure or rates of initial wound closure [16].

### 4.6. Next Generations of Clinical Progenitor Cell-Based Cytotherapeutics and Derivatives: Improving Stability, Fighting Patient Infection, and Reducing Product Degradation

The extensive clinical experience set forth herein has enabled a constant challenging of the boundaries of cutaneous regenerative medicine care provision, with the development of adapted solutions and tools. Therefore, recent research directions under the Swiss progenitor cell transplantation program have comprised the development of ancillary components or processing methods, aiming mainly to fight infectious risks in burn patient populations, as well as to provide improved, stable, and safe cytotherapy-inspired derivative products. Key considerations shaping and orienting the next steps in therapy development have been cell source processing, clinical administration modalities, and the use of cell-derived cell-free biological materials, to cite only a few recent scientific and technical areas of focus [49,50,51,52,53,54,55]. It is noteworthy that many groups have lately been investigating cell sources similar to those discussed herein for therapeutic use, at various stages of theoretical work, applied research, or preclinical development [55,56,57,58,59]. Overall, it may be stated that holistic optimization is necessary for sound development and effective translational advancements leading to enhanced clinical success.

For the optimal illustration of the adopted past and present academic and parallel research directions in Lausanne, the various generations of investigated progenitor biological bandages are summarily listed hereafter, along with the corresponding stages of research or development and clinical use:

- First-generation PBBs: Collagen scaffolds seeded with viable and growth-capable dermal progenitor fibroblasts (i.e., clinical stage, multiple clinical trials, on-going) [15,16].
- Second-generation PBBs (PBI): Collagen scaffolds seeded with viable and growth-arrested (i.e., γ-irradiated) dermal progenitor fibroblasts (i.e., clinical stage, currently discontinued).
- Third-generation PBBs: Similar to the first generation, with addition of antimicrobial dendrimers, for combination of intended effects and management of the infectious risk (i.e., preclinical stage in large animal model) [60].
- Fourth-generation PBBs: Appropriate vehicle yielding temperature-stabilized non-viable dermal progenitor fibroblasts, for an off-the-shelf availability (i.e., development phase) [38].
- Fifth-generation PBBs: Appropriate vehicle yielding cell-derived cell-free and temperature-stabilized therapeutic extracts, for an off-the-shelf availability (i.e., development phase).

Overall, the development efforts mentioned hereabove have been deployed with the objectives of maximizing the scope of potential therapeutic gains procured by the intervention, augmenting the availability of treatments, and enhancing manufacturing quality [38]. While such modifications to historically implemented protocols require additional technical and regulatory work, numerous benefits have already been demonstrated in the past for widely available off-the-shelf and functional preparations, as mentioned previously for progenitor cell derivatives in topical semi-solid formulations [31,32].

In particular, the results of previous in vitro studies and preclinical or clinical applications have suggested that the retention of cellular viability or the presence of the original cellular structures was not required to conserve specific therapeutic functions [31,32,38,61,62,63,64,65,66,67,68]. Therefore and as mentioned above, further potential applications inspired by dermal progenitor cytotherapies comprise the topical use of cell-derived cell-free biological complexes (e.g., cell secretomes, exosome fractions) in cutaneous regenerative medicine [38]. Proteins associated with the cell secretome are mainly located extracellularly or in the cytoplasm, whereas exosomes are located mainly in the membrane, cytoplasm, and cytosol. While the former are implicated mainly in different metabolic, immune, and endocrine system-related pathways, the latter are mainly associated with endocytosis, cell junctions, other cell signaling pathways and platelet activation [62,63]. Therefore, further functional assessments of the various dermal progenitor cell-based derivatives may be undertaken in order to identify the optimal manufacturing process and regulatory pathway for novel topical regenerative medicine approaches. Overall, it should nonetheless be stated that the influence of starting biological material selection represents a critical factor within the sourcing and manufacturing process and should be the object of careful consideration, in view of potentially obtaining optimal therapeutic results.

### 4.7. Current Status of the Clinical Work around Progenitor Biological Bandages in Switzerland: Local Perspectives of Clinical Development

Building on the existence of multi-centric GMP manufacturing capacities of the considered dermal progenitor cell sources and on the previous validation by multiple regulatory agencies for clinical investigation of the corresponding cytotherapies, methodological updates are currently being implemented into the clinical work performed in the Lausanne burn center [5]. Following specific requirements of the current Swiss legal and regulatory landscape, the national regulator Swissmedic has officially authorized (i.e., in January of 2022, case file N°2020TpP1010) the continued investigational clinical use of PBBs (Table 2) [30]. In parallel to ongoing research on next-generation PBBs, this important approval has enabled the maintenance of such safe clinical practices within a clinical trial, with a broadening of the mid-term horizon for similar applications of alternative and locally designed allogeneic cytotherapies (e.g., for musculoskeletal disorders) in Switzerland [18,69,70,71]. Specific pathways applied to forthcoming PBB clinical evaluation in Lausanne correspond to a standardized transplant product clinical trial and marketing authorization process, wherein risk-benefit ratios and objective endpoints must be studied for therapy clinical validation.

In detail, cantonal ethical validation has been granted (i.e., CER-VD, case file BASEC-ID 2020-01873) for the mentioned upcoming clinical trial on PBBs in the context of the CHUV Priority Project Bru_PBB. This phase I/II interventional, prospective, and randomized monocentric clinical study (i.e., titled “Evaluation of the safety and effectiveness of PBBs in burn care”, trial identification number NCT05339490) will include at least 76 burn patients over the next five to ten years in two study arms (i.e., PBB application on second-degree burns and DSW). The objectives of this new clinical study, in addition to the authorized investigational use of PBBs, comprise the potential renewed demonstration of short-term and long-term cytotherapeutic care efficacy (e.g., bettering of wound re-epithelialization, scar appearance and color, skin elasticity, viscoelasticity, long-term extension/retraction potential, and pliability).

Overall, the presented case-study of PBBs is useful for the demonstration that with proper methodological devising and technical adaptation, specific cytotherapeutic interventions may evolve and persist despite drastic changes in the local healthcare ecosystem. Therein, process and therapy versatility may be usefully combined with the acquired hindsight and analysis of current global regulatory trends, to ensure that continued focus and driving forces locally animate the forefront of topical cytotherapeutic care.

## 5. Conclusions

The aim of this study was to set forth the consolidated clinical data, experience, and hindsight gathered over thirty years around the use of allogeneic dermal progenitor fibroblasts and derivatives for topical therapeutic applications. Such practices were set in the context of the current global cytotherapy ecosystem. Original data was provided for various clinical steps and issues addressed under the Swiss progenitor cell transplantation program since 1991 for cutaneous cytotherapeutic care provision. Along with the high versatility and robustness of primary progenitor fibroblasts used as biological starting materials, critical aspects of clinical safety were summarized herein for such allogeneic cells (i.e., treatment of vulnerable patients, with a high variability in patient profiles and clinical presentation). Three decades of clinical work, with the needs of over 300 patients addressed, have generated robust hindsight and technical know-how for cell-based and cell-derived therapy translation and transposition. Overall, this study covered the strong current and global interest in biological-based approaches to cutaneous regenerative medicine, with an orientation toward the clinical use of allogeneic cytotherapies based on dermal progenitor fibroblasts. Taken together, these current and historical elements may serve as tangible working bases for further optimization of local and modern translational pathways, for the provision of high-quality topical cytotherapeutic care.

## Figures and Tables

**Table 1:** Summary of registered randomized clinical trials around the world (i.e., completed or ongoing trials) on the homologous therapeutic use of allogeneic progenitor cells from cutaneous origin. The primary and secondary outcomes of the listed clinical trials are further summarized in Table S1. DSW, donor site wound; PBB, progenitor biological bandages.

Clinical Study Name	Clinical Study Design	Patient Population Size	Patient Demographics	Type of Wound	Product Used, Cell Types, Formulation, Delivery System	Type of Comparison Treatment	Mean Healing Time	Follow-Up Time	Study Completion Date
“The effects of acellular amniotic membrane loaded by cultured fetal fibroblast cells in split thickness skin wound healing”	Study Type: Interventional Allocation: Randomized Masking: Double-blinded Phase: Phase 1	10 (the patient is his own control)	12–60 years old Sexes: All No healthy volunteers accepted	DSW	Amniotic membrane seeded with fibroblasts; Acellular amniotic membrane; Temporary coverage	Amniotic membrane; vaseline gauze	12.1 ± 3.1 days	23 ± 5 days	June, 2015
“TWB-103 for adult patients with split-thickness skin graft donor site wounds” ^1^	Study Type: Interventional Phase: Phase 1 and 2 Allocation: Randomized Intervention Model: Parallel Assignment Masking: Triple (Participant, Investigator, Outcomes Assessor)	48 (24 controls)	20–65 years old Sexes: All No healthy volunteers accepted	DSW	TWB-103 add-on Tegaderm; Hydrogel seeded with fibroblasts; Temporary coverage	Placebo hydrogel; Tegaderm	From DSW creation to the first 100% re- epithelialization, D42 or earlier	1 year	7 May 2021
“Controlled comparison of a traditional dressing versus a biologic dressing composed of fetal fibroblasts and keratinocytes in association with a collagen matrix on skin donor sites (CICAFAST)”	Study Type: Interventional Phase: Phase 1 and 2 Allocation: Randomized Intervention Model: Crossover Assignment Masking: None (Open Label)	38 (the patient is his own control)	>18 years old Sexes: All No healthy volunteers accepted	DSW	Biological dressing CICAFAST; Bovine collagen matrix of 100 cm^2^ seeded with fibroblasts and keratinocytes; Temporary coverage	Paraffin gauze; Jelonet	Healing at D8 (or D11 or D15 if the healing is not completed)	6 months	16 November 2023 (estimated)
“Evaluation of the safety and effectiveness of progenitor biological bandages in burn care” ^1^	Study Type: Interventional Phase: Phase 1 and 2 Allocation: Randomized Intervention Model: Parallel Assignment Masking: Single (Participant)	76 (estimated)	Child, adult, older adult Sexes: All No healthy volunteers accepted	DSW	Progenitor biological bandages (PBB); Equine collagen matrix of 108 cm^2^ seeded with fibroblasts; Temporary coverage	Jelonet	Maximum of 15 ± 1 days	5 years	1 May 2023 (estimated)

^1^ It should be noted that clinical trials conducted in Switzerland and in Taiwan or Japan have been and are performed using the same therapeutic starting biological material (i.e., same cell source, FE002-SK2 cell type), in an effort to standardize manufacturing techniques and clinical practices [5].

**Table 2:** Summary of the clinical cytotherapeutic applications of cultured allogeneic dermal progenitor fibroblasts (i.e., viable cells at the time of application) in a specific pharmaceutical form (i.e., biological bandages) and in various therapeutic indications. In parallel to the clinical work performed in Switzerland, further investigative use of dermal progenitor fibroblasts under the framework of the Swiss progenitor cell transplantation program has been carried out in Asia for skin donor-site wounds (i.e., NCT02737748 trial, registered in 2016) and diabetic foot ulcers (i.e., NCT03624023 trial, registered in 2018). It is important to note that since 2009, following regulatory updates and transition to GMP processing, all human patients have been treated with viable dermal progenitor fibroblasts from the FE002-SK2 cell source. CHUV, centre hospitalier universitaire vaudois; ePBB, equine progenitor biological bandage; NA, non-applicable; PBB, progenitor biological bandage; PBI, progenitor biological bandage yielding γ-irradiated cells.

Type of Biological Bandage	Treatment Indication	Years of Clinical Application	Implicated Clinical Centers	Patients Treated (*n*)	Clinical Trials & Literature References
PBB	Primary burn wounds	2001–Present	CHUV, Lausanne, Switzerland	>100	NCT05339490 [15,16,30,33]
	Donor-site wounds	2001–Present	CHUV, Lausanne, Switzerland	>50	NCT05339490 [30]
	Chronic lower-limb ulcers	2001–2005	CHUV, Lausanne, Switzerland; Private medical practice, Switzerland	>15	[34]
PBI	Primary burn wounds	2013–2015	CHUV, Lausanne, Switzerland	22	NA (unpublished results)
	Donor-site wounds	2013–2015	CHUV, Lausanne, Switzerland	>10	NA (unpublished results)
ePBB	Traumatic wounds ^1^	2000–2003	Private veterinary practice, Switzerland	4	[35]

^1^ Veterinary patients (i.e., equines). It should be noted that cytotherapeutic care of two additional equine burn patients was planned with ePBBs under compassionate use at the time, yet clinical and logistical elements (i.e., need for repeated anesthesia, impossibility to maintain prolonged aseptic conditions in veterinary housing) rendered the treatment impossible.

**Table 3:** Overview of clinical applications of PBI and PBB constructs in adult and pediatric patient populations between 2013 and 2021 in the Lausanne burn center (i.e., for primary burn wounds or for skin donor site wounds). Extreme clinical cases where patients have received the highest recorded product doses are mentioned. NA, non-applicable; PBB, progenitor biological bandage; PBI, progenitor biological bandage yielding γ-irradiated cells; TBSA, total body surface area.

Year	Type of Biological Bandage		Number of Patients Treated (*n*)		Mean PBB Amount/Bandage Exchange Procedure (*n*)		Mean Total PBB Amount/Treated Patient (*n*)	
	PBB	PBI	Pediatric	Adult	Pediatric	Adult	Pediatric	Adult
2013	NA	40	1	1	20.0	20.0	20.0	20.0
2014	NA	350	9	1	15.7	10.0	36.7	20.0
2015a	NA	174	2	8	14.0	8.7	35.0	13.0
2015b	152	NA	5	4	11.9	11.3	21.4	11.3
2016	819	NA	8	9	12.8	30.9	36.8	58.3
2017 ^1^	1015	NA	6	9	17.9	34.8	41.7	85.0
2018 ^2^	830	NA	4	7	30.9	27.2	85.0	70.0
2019	492	NA	11	4	8.4	19.1	29.1	43.0
2020	415	NA	6	5	10.9	26.7	29.2	48.0
2021	164	NA	10	0	7.1	NA	16.4	NA

^1^ One adult patient with 90% TBSA burns received 320 PBBs (i.e., 34,560 cm^2^ in total) in 7 applications in 2017. ^2^ One pediatric patient with 35% TBSA burns received 290 PBBs (i.e., 31,320 cm^2^ in total) in 6 applications in 2018. It should be noted that 3–4 PBB applications are usually performed for a given patient.

## Data Availability

Data presented in this study are available upon reasonable request made in writing to the corresponding author. Clinical data are also made publicly available on the www.clinicaltrials.gov (accessed 12 September 2022) database for individual clinical trials.

## Supplementary Materials

The following supporting information can be downloaded at: https://www.mdpi.com/article/10.3390/pharmaceutics15010184/s1, Figure S1: Organizational and technical overview of the Swiss progenitor cell transplantation program; Figure S2: Illustrated technical workflows for progenitor cell manufacture and formulation into topical therapeutic products; Figure S3: Illustrated parametric workflow for progenitor cell sourcing, manufacture, and formulation; Figure S4: Characteristics of FE002-SK2 cells and PBB constructs; Figure S5: Evolutive cellular morphology of proliferating FE002-SK2 primary progenitor cells; Figure S6: Graphical overview of FE002-SK2 cell type characterization work; Figure S7: Illustrative photographic timelapse of the extemporaneous preparation of a PBB therapeutic unit; Figure S8: Illustrative photographic overview of progenitor cytotherapy in vitro processing and clinical PBB administration in the Lausanne burn center; Figures S9–S11: Photographic records of pediatric patient hand burns (i.e., various heat sources) treated with PBBs, including long-term follow-up; Figures S12–S15: Photographic records of geriatric refractory lower-limb ulcers treated with PBBs, including follow-up; Figure S16: Illustrated overview of PBB construct preparation; Figure S17: PBB lot reception and use in the operating theatre; Table S1: Primary and secondary outcomes of registered clinical trials on allogeneic cutaneous progenitor cells; Table S2: Details on clinical protocols and clinical trials for PBBs; Table S3: Formulation details for various versions of progenitor biological bandages; Supplementary Document “PBB Monograph”: Cytotherapeutic product general presentation and technical information document.

## Abbreviations

ATMP	advanced therapy medicinal product
cATMP	combined advanced therapy medicinal product
CHUV	centre hospitalier universitaire vaudois
CTMP	cell therapy medicinal product
DSW	donor-site wound
FDA	US Food and Drug Administration
FE002-SK2	primary skin-derived progenitor cell type
GLP	good laboratory practices
GMP	good manufacturing practices
GTMP	gene therapy medicinal product
IB	investigator’s brochure
IMPD	investigational medicinal product dossier
MSC	mesenchymal stem cell
NCT	clinical study unique identification code
PBB	progenitor biological bandage
PMDA	Pharmaceuticals and Medical Devices Agency
TEM	transmission electron microscopy
TEMP	tissue engineered medicinal product
TFDA	Taiwan Food and Drug Administration
UK	United Kingdom
USA	United States of America
