# Supplementary material for: Retrospectives on Three Decades of Safe Clinical Experience with Allogeneic Dermal Progenitor Fibroblasts: High Versatility in Topical Cytotherapeutic Care
Source: Pharmaceutics. 2023 Jan 4;15(1):184. doi: 10.3390/pharmaceutics15010184 (PMC9866885; doi:10.3390/pharmaceutics15010184)
Supplement: Supplementary file 1 [file pharmaceutics-15-00184-s001.zip › PBB Monograph.pdf]

Version 07/2022/LALA

PROGENITOR BIOLOGICAL BANDAGES

Biological progenitor fascia

This monograph constitutes a technical norm for the preparation, the control, and the clinical therapeutic use of progenitor biological bandages ("PBB") in cutaneous regenerative medicine. The contents of this monograph do not exclude or supersede the use of alternative processes and methods for the preparation and control of the PBB cytotherapeutic product, as agreed upon with the competent Health Authority.

1. PBB DEFINITIONS & PRODUCT DESCRIPTION

Progenitor biological bandages ("PBB") are a form of ready-to-use cytotherapeutic product for clinical use incorporating cultured primary mammalian cells in viable form. In an allogeneic cytotherapeutic setting, PBB constructs are intended for topical homologous use in cutaneous regenerative medicine, for the management of diverse acute or chronic cutaneous wounds (e.g., burns, ulcers, skin donor-site wounds). In their final packaging, PBB constructs are composed of the following:

- Viable fibroblastic progenitor cells (FPC, e.g., FE002-SK2 primary cell type) derived from dermis, which act as the cellular active pharmaceutical ingredient (API); stored at –196°C to –160°C in liquid nitrogen.
- Lyophilized acellular equine collagen sheet scaffolds (e.g., 9 cm × 12 cm dimensions, 2 mm thickness), which act as the cell delivery scaffold (excipient); provided as a sterile and CE-marked medical device.
- Isotonic and buffered liquid transport medium, which acts to maintain appropriate physico-chemical conditions for the cellular API within the prepared construct (excipient); provided as a sterile medicinal product.

The API composing the PBB constructs consists in cryopreserved cultured mammalian primary diploid progenitor cells of fibroblastic nature at defined cell population doubling levels or *in vitro* passage levels. Primary progenitor cells used as the API in the PBB constructs are obtained under a defined progenitor cell transplantation program (i.e., organ donation from a recruited, qualified, and tested donor) and are manufactured *in vitro* following optimized, defined, and validated technical specifications in a GMP multi-tiered cell banking system (i.e., Parental Cell Bank, Master Cell Banks, Working Cell Banks) following best practices and the state-of-the-art. The cellular API is conditioned appropriately as a cell suspension with the use of excipients in view of long-term storage (e.g., cryopreservation medium). The considered API materials cumulatively comply with Ph. Eur. general monographs 2034 "*Substances for pharmaceutical use*", 2619 "*Pharmaceutical preparations*", and 1483 "*Products with risk of transmitting agents of animal spongiform encephalopathies*". Primary FPC APIs are available as individual cryopreserved vials of cells to be extemporaneously reconstituted and used to seed the collagen scaffolds to form the PBB constructs. The viable dermal FPCs adhere to the collagen scaffolds during an *in vitro* incubation period (i.e., 18–24 hours) after the cell seeding procedure. The PBB constructs are intended to be appropriately and extemporaneously reconstituted by qualified personnel within an appropriate GMP-compliant infrastructure and quality system. The final form of the PBB product is tested for quality following defined methods, targets, and acceptance criteria prior to liberation of the manufactured lot and transfer to the clinic.

In addition to the specified requirements of the present monograph, specific requirements pertaining to API production, qualification testing, characterization testing, safety testing, and PBB product lot release testing may be further included in individual specific monographs, such as a cell and tissue monograph as defined by the EDQM "Guide to the quality and safety of tissues and cells for human application". The present monograph generally refers to Regulation EC 1394/2007 on advanced therapy medicinal products, Directive 2001/83/EC on the Community code relating to medicinal products for human use, the EMA Guideline "Human cell-based medicinal products" (EMEA/CHMP/410869/2006), Commission Directive 2003/94/EC laying down the principles and guidelines of good manufacturing practice in respect of medicinal products for human use and investigational medicinal products for human use, and the EC "Guidelines on good manufacturing practice specific to advanced therapy medicinal products" (GMP ATMP vol. 4 - Part IV). The objective of the present monograph is to standardize the preparation, testing, and clinical use of PBBs, to ensure high quality, safety, and efficacy of cytotherapeutic care.

2. PBB INTENDED USES & THERAPEUTIC INDICATIONS

PBBs were designed to be clinically applied as early and temporary bioresorbable first covers on second-degree (i.e., superficial and deep) burn wounds, for the promotion of cutaneous wound closure, for the prevention of hypertrophic scarring, and for the reduction of the need for corrective surgeries. Historically, PBBs were also clinically applied as temporary covers on refractory lower-limb cutaneous ulcers, for the promotion of chronic wound healing. Current investigational cytotherapeutic uses of PBBs (i.e., defined as a standardized transplant product) in Switzerland (i.e., as authorized by the Health Authority and the Therapeutic Products Agency) comprise the following:

- Topical application as early and temporary first covers on skin graft donor-sites (e.g., in the case of skin harvest for autologous treatment of third-degree burn wounds).
- Topical application as early and temporary first covers on second-degree (i.e., superficial and deep) burn wounds, for the promotion of cutaneous wound closure, for the prevention of hypertrophic scarring, and for the reduction of the need for corrective surgeries.

Additionally, in extreme clinical cases (i.e., third-degree to fourth-degree burn wounds), PBBs may potentially be used under medical emergency use as early and temporary first covers on severe burn wounds, for the preparation of the wound bed for subsequent autologous skin grafting.

3. PBB THERAPY CONTRA-INDICATIONS

The following contra-indications have been specified for the therapeutic use of PBBs:

- Vitally unstable patient.
- Wound microbiological colonization.
- Wound microbiological infection.
- Bleeding wounds.
- Profusely exudating wounds.
- Known allergy or known hypersensitivity of the patient to a product component.

No data is currently available about the use of PBBs in pregnant and in lactating women.

4. PBB PRODUCTION METHODS & PBB LOT TESTING

4.1. PBB PRODUCT ATTRIBUTES

The following product attributes have been specified for the current version of the PBB cytotherapeutic product:

© Swiss progenitor cell transplantation program

- The collagen scaffolds composing the volume of PBBs are characterized by planar dimensions of 9 cm × 12 cm (i.e., alternative planar dimensions available) and a thickness of 2 mm.
- Each 9 cm × 12 cm PBB construct is seeded with a cell suspension containing 5 × 10^5 viable dermal FPCs, extemporaneously initiated from a dedicated progenitor WCB.
- All starting materials, raw materials, and ancillary materials used during the manufacture of the PBB construct lot are tested or are certified for the absence of contaminants.
- PBBs are manufactured on-demand in GMP conditions in lots of 4 constructs at minimum following the clinical prescription.
- Final PBB construct lot quality is evaluated based on *in vitro* cellular morphology, homogeneity of construct cell seeding, physical integrity of the construct and of its packaging materials (i.e., primary and secondary packaging).
- PBBs are not submitted to any form of terminal sterilization. The aseptic processing of the PBB construct lot should therefore guarantee the absence of adventitious agents (e.g., bacteria, fungi, mycoplasma, endotoxins) in the liberated lot.

4.2. QUALITY SYSTEM & PBB PRODUCTION ENVIRONMENT

The PBB manufacturing steps should be carried out under an appropriate quality system, usually a pharmaceutical quality system, with appropriately implemented quality risk management procedures, as described in ICH Q9 on quality risk management (EMA/CHMP/ICH/24235/2006) and in ICH Q10 on pharmaceutical quality systems (EMA/CHMP/ICH/214732/2007).

PBB product lot manufacturing is carried out in an appropriate environment under conditions minimizing the risk of contamination of all the materials by adventitious agents. The PBB manufacturing steps including open container manipulations are performed in a dedicated environment, for which the air quality is specified, validated, and monitored. Therein, and in the absence of microbiological contaminant removal or inactivation steps (e.g., terminal sterilization), the manufacturing environment must conform to GMP Grade A, as defined in Directive 2003/94/EC (i.e., with particle enumeration and microbial colony counts), with a background environment conforming at least to Grade B, if the primary manufacturing environment is not isolated.

4.3. MANUFACTURING EQUIPMENT & MATERIALS

All of the equipment and materials used in the manufacturing process of PBBs are specifically designed and appropriately maintained to suit the intended purpose and to minimize any hazard to the patients and to the manufacturing personnel. All of the critical equipment and devices are identified, qualified, regularly inspected, and preventively maintained, as appropriate. Key and critical process parameters in relation with the equipment and materials must be defined, along with acceptance margins and criteria, and are included in general and in specific risk analyses. In the case where the equipment, devices, or materials are determined to affect the critical processing or storage parameters (e.g., temperature, air pressure, particle counts, and microbial contamination levels), they must be appropriately identified and subjected to monitoring, including alerts, alarms, and corrective actions, if necessary, to detect and prevent any malfunctions or defects and to ensure that the critical parameters are always maintained within acceptable limits. Any equipment purposed with a critical measuring function is calibrated against a traceable standard, if available. Maintenance, servicing, cleaning, disinfection, and sanitation of all the critical equipment are performed regularly and are recorded.

The technical specifications for PBB manufacturing necessarily list all the critical materials (i.e., starting materials, raw materials, ancillary materials, conditioning materials), reagents, and consumables (i.e., contact-process consumables, non-contact-process consumables), along with their respective attributes. If applicable, the critical materials, reagents, and consumables must meet compendial requirements and/or documented specifications and the requirements of medical device regulations. Materials, reagents, and consumables exempt of animal-sourced components should be used where possible, provided that they are appropriately comparatively qualified and validated against historical specifications and available data.

Specifically, raw materials included in the manufacturing processes of PBB constructs are produced in appropriate qualified facilities under a recognized quality management system and are qualified. This aspect is important for the insurance of product critical quality attribute consistency (i.e., quality, safety, and efficacy). The use of antibiotics is avoided wherever possible during production activities. Control of raw material change or use of new raw material batches must be considered from a production perspective in light of Ph. Eur. general chapter 5.2.12. "Raw materials for the production of cell-based and gene therapy medicinal products".

4.4. RISK ASSESSMENTS

Appropriate multifactorial risk assessments are to be carried out, specifically concerning raw materials used in the manufacture of PBBs, to guarantee maximal quality, safety, and efficacy of the medicinal products. In general, the risk assessments are to be carried out following Ph. Eur. general chapter 5.2.12. for raw materials, and an appropriate HACCP approach may be applied to the manufacturing system as a whole. Risk analyses should be the basis of the working risk management plan and should be used to justify the product development and evaluation plans. Therefore, risk analysis and risk minimization activities govern the whole product lifecycle, from the product development to the post-market risk management (e.g., pharmacovigilance plan) activities.

4.5. PBB CONSTRUCT MANUFACTURING PROCESS

4.5.1. Thawing of Cryopreserved Progenitor Fibroblasts

The initiation of the cryopreserved vials of cellular API for direct seeding of the PBB construct scaffold is performed according to the following steps:

- Preparation of all of the necessary materials for the defined number of PBBs to be manufactured in the production suite.
- Selection of the cryopreserved API vials to use for PBB lot manufacture.
- Retrieval of the cryopreserved API vials from cryogenic storage, with update of the corresponding storage logs.
- Transfer of the frozen API vials to the production suite on dry ice.
- Thawing of the frozen API vials in a dry bath set at 37°C.
- Transfer of the thawed cell suspension to the class A zone.
- Dropwise transfer of the thawed and homogenized cell suspension into a centrifuge tube containing 14 mL of warm (37°C) cell culture medium.
- Centrifugation of the diluted cell suspension at 230 × *g* for 10 minutes at ambient temperature.
- Aseptic removal of supernatants.
- Resuspension of cell pellets using warm cell culture medium.
- Pooling of cell suspensions in an appropriate container and homogenization of the pool. Isolation of 3 × 0.5 mL of pooled suspension samples for cell viability control, total and viable cell counts determination.
- Determination of total and viable cell counts by microscopic enumeration. Verification that relative cell viability is ≥80% at this stage.
- Aseptic transfer of the cell suspension to an appropriate sterile container and dilution of the cell suspension using warm cell

© Swiss progenitor cell transplantation program

culture medium so that the final cell concentration reaches 10^5 total cells/mL.

4.5.2. PBB Scaffold Mechanical Preconditioning & Cell Seeding

Before seeding of the cell suspension on the collagen scaffold, a mechanical preconditioning step is required, as described hereafter. Also see *Figure S16* for the step-by-step illustration of the process.

- Opening of the scaffold packaging and aseptic transfer of the collagen sheets to individual Petri dishes. The most porous or hydrophilic side of the scaffold should face up in the culture dishes. Beware of static electricity build-up, which can cause the scaffolds to stick to culture vessels or to instruments.
- Indentation of the collagen scaffolds using sterile tweezers in series of eight considering the long side and in series of four considering the short side of the scaffold.
- Homogenous distribution of 5 mL of cell suspension (i.e., 5×10^5 cells) onto the 9 cm × 12 cm collagen scaffold in a crosswise pattern.
- Distribution of the cell suspension over the whole preconditioned surface of the collagen scaffold.

4.5.3. PBB Construct Lot Preparation & Liberation

For optimal cell survival and cell colonization of the scaffold, the latter requires a minimal period of incubation immediately after the cell seeding step, as follows:

- Aseptic addition of 25 mL of warm (37°C) cell culture medium to the edge of each dish containing a seeded collagen scaffold.
- Transfer of the dishes to the humidified incubator set at 37°C and 5%–10% CO_2.
- Incubation of the dishes for at least 18–24 hours.
- Removal of the dishes from the incubator.
- Macroscopic and microscopic inspection of the dishes for the exclusion of any obvious signs of contamination or observable defects in the constructs.
- Removal of the cell culture medium and liquid retention sample isolation.
- Washing of the PBB constructs with D-PBS to eliminate ancillary material traces and cell debris.
- Addition of 4 mL of isotonic and buffered transport medium to the surface of each construct for hydration maintenance.
- Sealing of the dishes, conditioning of the PBB lot in a sealed plastic bag, and labelling for transport to the clinic.
- Establishment of product lot documentation and release report.
- Transfer of the product lot to the clinic (<2 hours after conditioning).

If justified, alternative manufacturing methods may be designed, validated, and used for the manufacture of PBB constructs, provided that the critical safety and quality attributes of the obtained construct lots can be demonstrated as being equivalent. Therefore, the general principles of ICH Q5E "Biotechnological/biological products subject to changes in their manufacturing process: Comparability of biotechnological/biological products" (CPMP/ICH/5721/03) may be followed.

4.6. PBB CONSTRUCT LOT TESTING

Appropriate testing strategies are implemented following the general and specific risk analyses performed for critical processes within the manufacture of a PBB construct lot. For PBB lot testing purposes, the manufacturing steps comprised between API cell vial initiation and conditioning of the finished PBB construct lot are taken into account. All the tests are validated and performed under appropriate quality systems and standards (e.g., ISO 17025, GLP conditions), following ICH Q2 R1 "Validation of analytical procedures: Text and methodology" (CPMP/ICH/381/95). The testing scheme comprises the following:

- **Cell enumeration upon API thawing.** Nucleated cell count determination, as well as relative cellular viability are determined upon cellular API initiation according to Ph. Eur. method 2.7.29. "*Nucleated cell count and viability*".
- **Batch descriptive analysis.** Batch descriptive analysis is performed after construct incubation and after conditioning and is recorded, including construct structural integrity maintenance and integrity of primary/secondary product packaging.
- **Sterility testing.** Test-materials harvested at the time of API initiation, after construct incubation, and during final construct conditioning comply with the test for sterility, following Ph. Eur. method 2.6.1. "*Sterility*" and ICH Q5D or the Ph. Eur. method 2.6.27. "*Microbiological examination of cell-based preparations*" or Ph. Eur. method 5.1.6. "*Alternative methods for control of microbiological quality*".
- **Mycoplasma testing.** The test-materials comply with the test for mycoplasmas following Ph. Eur. method 2.6.7. "*Mycoplasmas*".
- **Bacterial endotoxins.** The test-materials comply with the test for bacterial endotoxins following Ph. Eur. method 2.6.14. "*Bacterial endotoxins*" or Ph. Eur. method 2.6.8. "*Pyrogens*".

The necessary critical quality attributes and quality specifications are determined for the final form of the PBB construct. The specifications and acceptance criteria are defined following ICH Q6B "Note for guidance on specifications: Test procedures and acceptance criteria for biotechnological/biological products" (CPMP/ICH/365/96).

5. FINISHED PBB CONSTRUCT LOT PACKAGING & PRESENTATION

PBB construct lots are provided to clinicians as ready-to-use therapeutic products, which are transported at ambient temperature to the operating theatre. Each PBB construct is individually conditioned in a sterile and sealed tissue-culture Petri dish with transport medium. A product lot is conditioned in a sterile and sealed plastic bag. The minimal lot size is of 4 PBB constructs, sufficient for 432 cm^2 of lesions to treat. Also see *Figure S17* for an illustration of a conditioned PBB lot and the adequate methods for PBB construct handling and application.

6. WARNINGS

The following warnings have been specified for PBB construct lots:

- PBBs are single-use products which are to be clinically applied on the patient within 8 hours of lot liberation from the GMP manufacturing facility.
- PBB constructs must not be frozen and should be discarded if they are not used for the specified patient or if they are not used within the specified validity period.
- A PBB construct lot which has been temporarily stored at temperatures <5°C or >45°C should not be used and should be discarded.
- A PBB construct delivered in a container with compromised structural integrity, or which is leaking, should not be used and should be discarded.
- PBBs should be deconditioned and applied by trained medical personnel in aseptic conditions in the operating theatre.
- PBBs should be manipulated cautiously to avoid construct structural ruptures. The PBB constructs may be cut and moulded to individual wound surfaces in the operating theatre by the attending physicians.
- PBB constructs should not be repositioned once applied on the wound.

© Swiss progenitor cell transplantation program

- Following application, PBB product residues are to be washed away with an appropriate irrigation solvent during standard wound care and normal bandage exchange procedures.
- Antiseptic preparations may be applied during standard wound care, but should be appropriately rinsed away before PBB product applications, to avoid interferences with the cellular API.
- Observed adverse reactions to the PBB products should be documented and should be notified to the product manufacturer.

7. PRECAUTIONS

The following precautions have been specified for PBB construct lots:

- PBBs should be stored at ambient temperature in the operating theatre between delivery and application, and away from high-intensity light sources.
- PBBs should be applied on the wounds as soon as possible following the delivery to the operating theatre.
- PBBs should be manipulated slowly and gently, to avoid construct ruptures.
- PBBs should be handled, cut, and applied using aseptic techniques.
- Applied PBB construct residues should be washed off within 4 days after the application.

8. ADVERSE REACTIONS

Adverse reactions should immediately be reported to the product manufacturing unit and the responsible physician. With regard to side effects directly related to the application of PBB constructs, two decades of clinical use have not yielded any evidence or observations pertaining to specific and confirmed treatment-related adverse reactions or events. Side effects inherent to the burn trauma itself may generally be observed (e.g., wound infections, pain, prolonged bleeding, pneumonia, urinary tract infections, punctiform keratitis, urticaria, upper respiratory tract infections) during the course of the treatment. Overall, few and non-severe adverse reactions have been reported to date following topical administration of PBB constructs to adult and pediatric burn patients in the Lausanne University Hospital – CHUV (i.e., >150 patients since 2001).

9. INSTRUCTIONS FOR USE OF PBB CONSTRUCTS

9.1. CLINICAL PBB CONSTRUCT APPLICATION

The PBB constructs may be applied directly after patient stabilization, cleaning, and debridement of the burn site/wound or at any appropriate subsequent timepoint. The therapeutic planification is defined by attending physicians, taking into account the minimal 18-hour notice period necessary for the manufacture of the PBBs (i.e., cellular API initiation and cell colonization of the collagen scaffolds under incubation). In practice, the first PBB lots are usually applied 24–48 hours after the patient is admitted to the burn center. Thereafter, PBB application procedures are performed every 2–4 days (i.e., maximal continuous wound coverage of 4 days) after medical re-evaluation of the wounds and for a maximum of 4 serial applications in current clinical protocols. Also see *Figure S17* for an illustration of a conditioned PBB lot and the adequate methods for PBB construct handling and application. For the clinical application of the PBB constructs, the attending clinician team should follow the following procedure:

- Showering of the patient.
- Debridement and wound bed disinfection according to the standard clinical protocols.
- Cleaning of all traces of disinfecting agent from the wounds.
- Photographic documentation of the wound bed appearance before treatment application, including a ruler.
- Opening of the PBB container and packages, removal of the PBB constructs from their dishes using two sterile forceps by holding both upper corners of the construct.
- If necessary, aseptically cut the PBB construct using surgical scissors.
- Application of the constructs to the patient, overlapping them where necessary, and with moulding to the specific topography of the wounds. Once an individual PBB construct is in place, it should not be displaced or moved, to avoid rupture of the construct.
- Evacuation of any trapped air bubbles from under the constructs, using very gentle manual massaging of the construct using the forceps or gloved fingertips.
- Photographic documentation of the wound bed appearance after the PBB construct application has ended.
- Once all the PBB constructs are placed, they are overlain serially with Jelonet® gauze and cotton gauze before standard bandages are applied.

9.2. PBB CONSTRUCT EXCHANGE PROCEDURES & COURSE OF THE TREATMENT

For the successive PBB construct application procedures, the attending clinician team should proceed as follows every 2–4 days, as necessary:

- Opening of the outer layers of the bandages until the biodegraded PBB construct remnants are apparent.
- Photographic documentation of the wound bed appearance with the remaining constructs.
- Removal of all of the construct remnants by irrigation and washing.
- Sampling for bacteriology analysis in case of suspicion of wound colonization or infection, followed by showering of the patient.
- Photographic documentation of the wound bed appearance without the constructs, including a ruler.
- Application of the necessary amounts of new PBB constructs.
- Repetition of the PBB construct applications, if needed, until sufficient skin closure is attained.

During the course of the treatment, the attending physician and the responsible physician will evaluate the situation regularly, with great attention being paid to the following points:

- The treatment duration with PBB constructs depends on the wound epithelialization and scar assessment, as evaluated by the responsible physician.
- Topical corticosteroids may be applied if necessary (e.g., in the case of tissue granulation) and following prescription by the responsible physician.
- At any phase of the treatment, the presence of severe local infection should prompt immediate PBB treatment discontinuation and thorough showering of the patient, according to the decision of the responsible physician.
- Applications of PBBs should be discontinued in the case of absence of skin closure after 2 weeks of treatment, according to the decision of the responsible physician, and a skin graft should be planned accordingly.
- All episodes of treatment with PBB constructs should be documented.
- The date, number of PBB constructs, and the zones of application should be recorded.
- During PBB treatment, usual procedures should be followed as for all burn patients, comprising minimal hospitalization periods, protection of newly formed cutaneous structures, daily massages as soon as structurally supported by the skin, and application of silicone pressure garments, if needed.

© Swiss progenitor cell transplantation program

PBB constructs will stay in contact with the wound for 2–4 days in the case of burn wound treatment or for up to five days in case of skin graft donor-site care. In general, a PBB treatment duration of maximum two weeks is used until the autologous skin grafting (if necessary) should be considered.

9.3. CLINICAL ENDPOINTS & EXPECTED THERAPEUTIC EFFECTS OF PBB CONSTRUCTS

During the course of the treatment with PBB constructs, regular re-assessment of the evolution of the wound bed by the attending clinician team and general evaluation of the patient should lead to formal evaluation of the effects of the treatment. Primary and secondary objective clinical endpoints relative to safety and therapeutic performance, such as the following, may be used for this evaluation:

- Days of treatment necessary to obtain satisfactory wound re-epithelialization, existence of a wound healing drive.
- Length of patient hospitalization, in ICU and in maintenance care.
- Wound healing rates and evolutive wound TBSA percentages.
- Quantity and surface of wounds requiring autografts.
- Pain questionnaires.
- Quantity of PBB constructs applied during the treatment.
- Scar management and duration thereof.
- Long-term assessment of skin functionality, expressed by skin mobility and contracture.
- Long-term skin quality and sensory modifications (e.g., Vancouver scar scale, erythema and melanin indexes).
- Number and nature of infections.

With regard to therapeutic performance and safety of application of the PBB constructs, the following observations have been made:

- The healing outcomes of treatments with PBB constructs are at least as good as other standard treatments (e.g., AQUACEL® Ag + or Jelonet® coverages).
- PBB treatment leads to total recovery of mobility (e.g., hands and fingers) and restoration of skin pigmentation.
- Application and removal of PBBs are painless and do not inherently require anaesthesia.
- PBB constructs alleviate the risk of hypertrophic scar formation, retraction, and secondary breakdown of healed surfaces.
- PBBs are microbiologically safe and pose no inherent risk of colonization or infection by virologic, bacterial, and fungoid agents.
- No additional fixation devices (e.g., glue, staples, stitches, silicones) are needed, and ease of application on all anatomical sites is evident.
- The use of progenitor cells alleviates the risk of immunological rejection of the treatment by the patient.
- PBBs induce no observable inflammatory reaction, no secretion, and no allergy.
- PBBs possess no inherent tumorigenic properties.

Patient eligibility and treatment necessity must be evaluated, based on the clinical case specificities, by the attending physician team before considering the therapeutic use of PBB constructs. Once the use of PBBs is validated, the adequate quantity of constructs is ordered from the GMP production unit. Validation of treatment initiation requires the following steps:

- The responsible physician evaluates the need for PBB construct application (e.g., second-degree burn lesions, >10%–20% TBSA wounds, extensive skin graft donor-sites).
- Ruling out of contra-indications for PBB construct application, and verification that no incompatibilities with the constructs are present during the treatment.
- Performance of bacteriological investigations (i.e., swab tests) of the wound beds to exclude colonization or infection if suspicions of such cases arise and are substantiated by several combined criteria (e.g., fever, specific odour, elevated inflammatory markers).
- Planning of the therapeutic applications of PBB constructs in accordance with the care schedule of the patient and the production capabilities of the GMP manufacturing unit. Ideal application of PBB constructs occurs after patient showering, debridement, and disinfection of the wounds, in the operating theatre.
- Ordering of the adequate quantity of PBB constructs for initial patient wound care.
- Organization of the delivery of the subsequent PBB construct lots with the GMP manufacturing unit according to the defined therapeutic calendar.

10. PBB CONSTRUCT LOT LABELLING

The following labelling items and product lot accompanying documentation items have been specified for PBB constructs:

- Product name, reference, and unique lot number.
- Name of the patient (i.e., PBBs are manufactured and destined for defined patients).
- Date and time of the manufacture start, end, and of the final product liberation.
- Product expiry date and time.
- Name of the attending/prescribing physician.
- Product transport conditions.
- Key product specifications.
- Instructions for appropriate product clinical application.
- Manufacturing lot liberation report.
- Manufacturer identification and contact details.
- Instructions for reporting serious adverse reactions and/or events.
- Instructions on how to dispose of unused or expired products.

11. PBB PRODUCT STORAGE & TRANSPORT

PBBs are extemporaneously manufactured on-demand following the medical prescription for a specific patient and are therefore not designed to be stored in their final form. PBBs are transported at ambient temperature (i.e., 15°C–25°C) in an isotherm and temperature-monitored transport container to the operating theatre. Low (i.e., <5°C) and high (i.e., >40°C) temperatures should be avoided during product handling and transport. The conditioned product lot should be protected from shocks and from direct exposure to strong light sources.

12. PBB PRODUCT SHELF-LIFE

The PBB shelf-life or validity period may be determined following ICH Q5C "Stability testing of biotechnological/biological products" (CPMP/ICH/138/95).

The shelf-life is the time-period during which the quality of the product remains within the specified limit thresholds. The shelf-life is the time-period during which the quality, safety, and efficacy of the product are demonstrated as being maintained. The product validity period after manufacture is specified and the final product should not be stored before application in the operating theatre for a longer period than specified (e.g., >8 hours).

13. CLINICAL SURVEILLANCE

The clinical use of PBBs must be subjected to surveillance in the framework of an appropriate pharmacovigilance plan, including reporting of possible adverse events and reactions associated with their use. Establishment of a centralized reporting system and of a dedicated register are warranted.

© Swiss progenitor cell transplantation program

Surveillance is performed in accordance with the requirements of Directive 2004/23/EC, Directives 2006/17/EC and 2006/86/EC, and of EMEA/CHMP/96268/2005.

14. AVAILABLE CLINICAL EVIDENCE ON PBB

The PBB construct manufacturing processes described herein have been validated at semi-industrial scales in GMP settings, with the demonstration that produced cytotherapeutic products fulfil the safety and quality standards for therapeutic use in human subjects. Described processes correspond to the current state-of-the-art, have been published in peer-reviewed scientific literature, and have been implemented in clinical use for over two decades in university hospital settings and in multiple clinical trials, as notably follows in selected literature abstracts:

- **Hohlfeld *et al.*, Lancet, 2005**: *Autologous skin-grafting is the gold standard for treatment of deep second and third degree burns. Available bioengineered skin products also necessitate this two-step surgical procedure. Therefore, we developed fetal skin constructs to improve healing of such degree burns. A bank of fetal skin cells was developed from one organ donation (4 cm^2 of skin allowing the preparation of several million three-dimensional skin constructs, 9×12 cm, on native horse collagen). Successive fetal constructs were applied to eight patients at every change of dressing during 1–3 weeks in an outpatient setting. Complete closure was rapid (mean 15.3 days [SD 5.5]) with little hypertrophy of new skin and no retraction seen. This simple technique provided complete treatment without auto-grafting, showing that fetal skin cells might have great potential to treat burns and eventually acute and chronic wounds of other types.*

- **Ramelet *et al.*, Experimental Gerontology, 2009**: *Engineering of fetal tissue has a high potential for the treatment of acute and chronic wounds of the skin in humans as these cells have high expansion capacity under simple culture conditions and one organ donation can produce Master Cell Banks which can fabricate over 900 million biological bandages (9×12 cm). In a Phase 1 clinical safety study, cases are presented for the treatment of therapy resistant leg ulcers. All eight patients, representing 13 ulcers, tolerated multiple treatments with fetal biological bandages showing no negative secondary effects and repair processes similar to that seen in 3rd degree burns. Differential gene profiling using Affymetrix gene chips (analyzing 12,500 genes) were accomplished on these banked fetal dermal skin cells compared to banked dermal skin cells of an aged donor in order to point to potential indicators of wound healing. Families of genes involved in cell adhesion and extracellular matrix, cell cycle, cellular signaling, development and immune response show significant differences in regulation between banked fetal cells and those from banked old skin cells: with approximately 47.0% of genes over-expressed in fetal fibroblasts. It is perhaps these differences which contribute to efficient tissue repair seen in the clinic with fetal cell therapy.*

- **De Buys Roessingh *et al.*, Journal of Regenerative Medicine, 2015**: *A decade ago, a study was reported on the management of pediatric burns using cell therapy and foetal skin progenitor cells. We now present follow-up on the patients in that original clinical study. We describe the regulatory requirements for clinical research, specifically that research conducted in a hospital environment and involving human cell and tissue based products. Also, how these requirements have changed since 2007 and how these changes might adversely affect burn victims and their clinical care pathways in the future will be addressed in this report.*

- **Al-Dourobi *et al.*, Pharmaceuticals, 2021**: *Progenitor Biological Bandages (PBB) have been continuously applied clinically in the Lausanne Burn Center for over two decades. Vast translational experience and hindsight have been gathered, specifically for cutaneous healing promotion of donor-site grafts and second-degree pediatric burns. PBBs constitute combined Advanced Therapy Medicinal Products, containing viable cultured allogeneic fetal dermal progenitor fibroblasts. Such constructs may partly favor repair and regeneration of functional cutaneous tissues by releasing cytokines and growth factors, potentially negating the need for subsequent skin grafting, while reducing the formation of hypertrophic scar tissues. This retrospective case-control study (2010–2018) of pediatric second-degree burn patients comprehensively compared two initial wound treatment options (i.e., PBBs versus Aquacel® Ag, applied during ten to twelve days post-trauma). Results confirmed clinical safety of PBBs with regard to morbidity, mortality, and overall complications. No difference was detected between groups for length of hospitalization or initial relative burn surface decreasing rates. Nevertheless, a trend was observed in younger patients treated with PBBs, requiring fewer corrective interventions or subsequent skin grafting. Importantly, significant improvements were observed in the PBB group regarding hypertrophic scarring (i.e., reduced number of scar complications and related corrective interventions). Such results establish evidence of clinical benefits yielded by the Swiss fetal progenitor cell transplantation program and favor further implementation of specific cell therapies in highly specialized regenerative medicine.*

15. ABBREVIATIONS

API	active pharmaceutical ingredient
CE	European conformity
CHUV	centre hospitalier universitaire vaudois
DMEM	Dulbecco's modified Eagle medium
D-PBS	Dulbecco's phosphate-buffered saline
EC	European Commission
EDQM	European Directorate for the Quality of Medicines and Healthcare
EMA	European Medicines Agency
FBS	fetal bovine serum
FPC	fibroblastic progenitor cells
GLP	good laboratory practices
GMP	good manufacturing practices
HACCP	hazard analysis critical control point
ICH	international council for harmonization
ICU	intensive care unit
ISO	international organization for standardization
MCB	master cell bank
PBB	progenitor biological bandage
PCB	parental cell bank
PDV	population doubling value
Ph. Eur.	European pharmacopoeia
SD	standard deviation
TBSA	total body surface area
US	United States of America
WCB	working cell bank

16. SCIENTIFIC LITERATURE & CLINICAL REFERENCE PAPERS

Scientific and clinical literature references relevant to the preparation and the clinical use of PBB constructs are provided hereafter in chronological order:

- Hohlfeld J, De Buys Roessingh AS, Hirt-Burri N, *et al.* (**2005**) Tissue engineered fetal skin constructs for paediatric burns. *Lancet* 366:840–842.
- De Buys Roessingh AS, Hohlfeld J, Scaletta C, *et al.* (**2006**) Development, characterization, and use of a fetal skin cell bank for tissue engineering in wound healing. *Cell Transplant.* 15:823–834.
- Quintin A, Hirt-Burri N, Scaletta C, *et al.* (**2007**) Consistency and safety of cell banks for research and clinical use:

© Swiss progenitor cell transplantation program

Preliminary analysis of fetal skin banks. *Cell Transplant.* 16:675–684.

- Hirt-Burri N, Scaletta C, Gerber S, *et al.* (**2008**) Wound-healing gene family expression differences between fetal and foreskin cells used for bioengineered skin substitutes. *Artif. Organs* 32:509–518.
- Applegate LA, Scaletta C, Hirt-Burri N, *et al.* (**2009**) Whole-cell bioprocessing of human fetal cells for tissue engineering of skin. *Skin Pharmacol. Physiol.* 22:63–67.
- Ramelet AA, Hirt-Burri N, Raffoul W, *et al.* (**2009**) Chronic wound healing by fetal cell therapy may be explained by differential gene profiling observed in fetal versus old skin cells. *Exp. Gerontol.* 44:208–218.
- Applegate LA, Weber D, Simon J-P, *et al.* (**2013**) Organ donation and whole-cell bioprocessing in the Swiss fetal progenitor cell transplantation platform. In: Saidi RF (ed.) *Organ donation and organ donors.* Nova Science Publishers, New York, NY, USA.
- De Buys Roessingh AS, Hirt-Burri N, Raffoul W, *et al.* (**2015**) A decade after fetal skin progenitor cell therapy in pediatric burn treatment. *J. Regen. Med.* 4:1.
- Abdel-Sayed P, Michetti M, Scaletta C, *et al.* (**2019**) Cell therapies for skin regeneration: An overview of 40 years of experience in burn units. *Swiss Med. Wkly* 149:w20079.
- Laurent A, Scaletta C, Michetti M, *et al.* (**2020**) Progenitor Biological Bandages: An authentic Swiss tool for safe therapeutic management of burns, ulcers, and donor site grafts. In: Turksen K (ed.) *Stem Cells and Good Manufacturing Practices.* Methods in Molecular Biology, vol 2286. Humana, New York, NY, USA.
- Laurent A, Lin P, Scaletta C, *et al.* (**2020**) Bringing safe and standardized cell therapies to industrialized processing for burns and wounds. *Front. Bioeng. Biotechnol.* 8:581.
- Al-Dourobi K, Laurent A, Deghayli L, *et al.* (**2021**) Retrospective evaluation of Progenitor Biological Bandage use: A complementary and safe therapeutic management option for prevention of hypertrophic scarring in pediatric burn care. *Pharmaceuticals* 14:201.
- Laurent A, Abdel-Sayed P, Scaletta C, *et al.* (**2021**) Back to the cradle of cytotherapy: Integrating a century of clinical research and biotechnology-based manufacturing for modern tissue-specific cellular treatments in Switzerland. *Bioengineering* 8:221.

© Swiss progenitor cell transplantation program

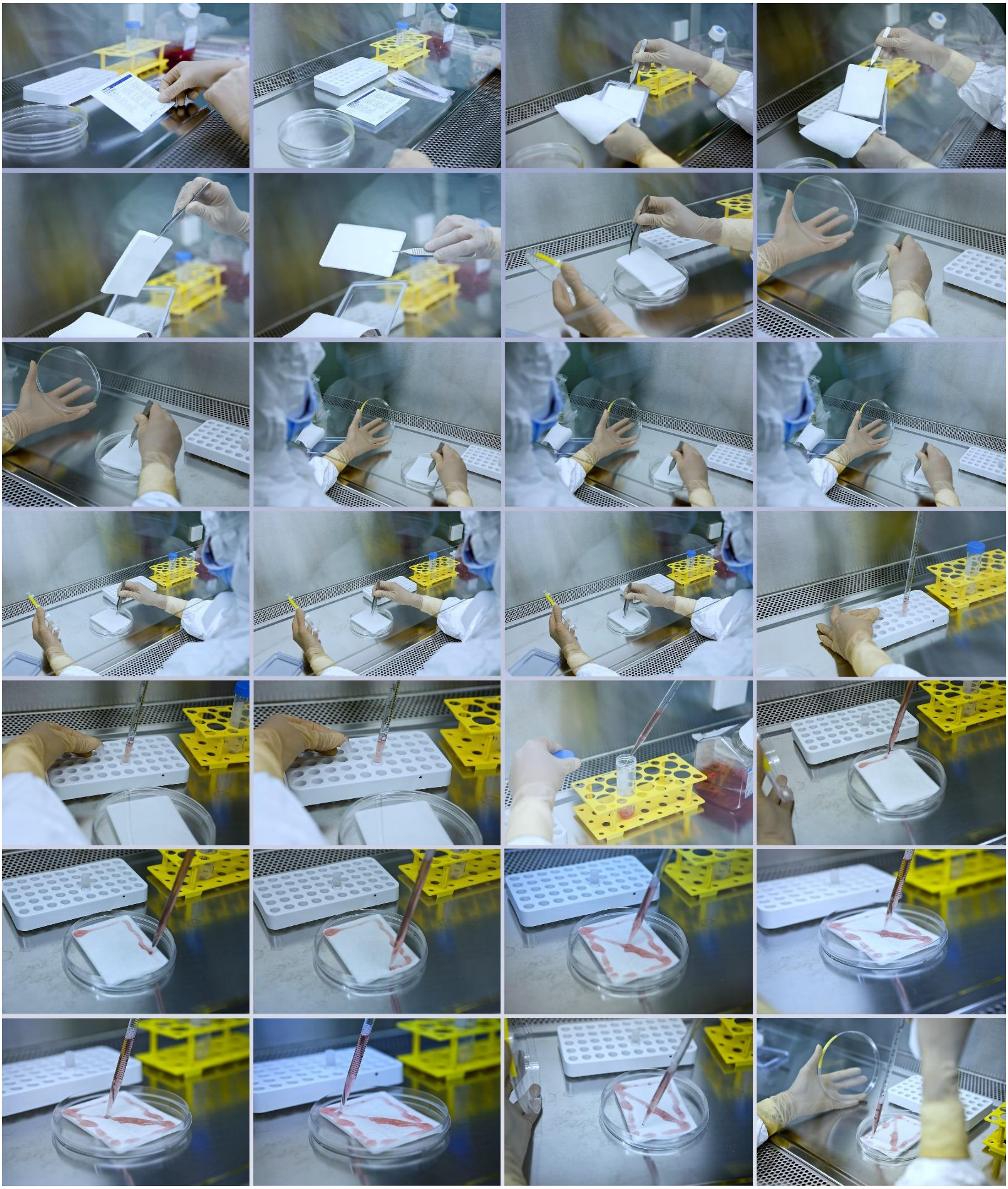

Figure S16. Illustrated stepwise overview of PBB construct preparation (*part 1/2*). Following unwrapping and mechanical preconditioning of the collagen scaffold using sterile tweezers, the therapeutic progenitor cell suspension is transferred consistently by manual pipetting, as to ensure the homogeneous repartition of the suspension over the entire surface of the scaffold.

© Swiss progenitor cell transplantation program

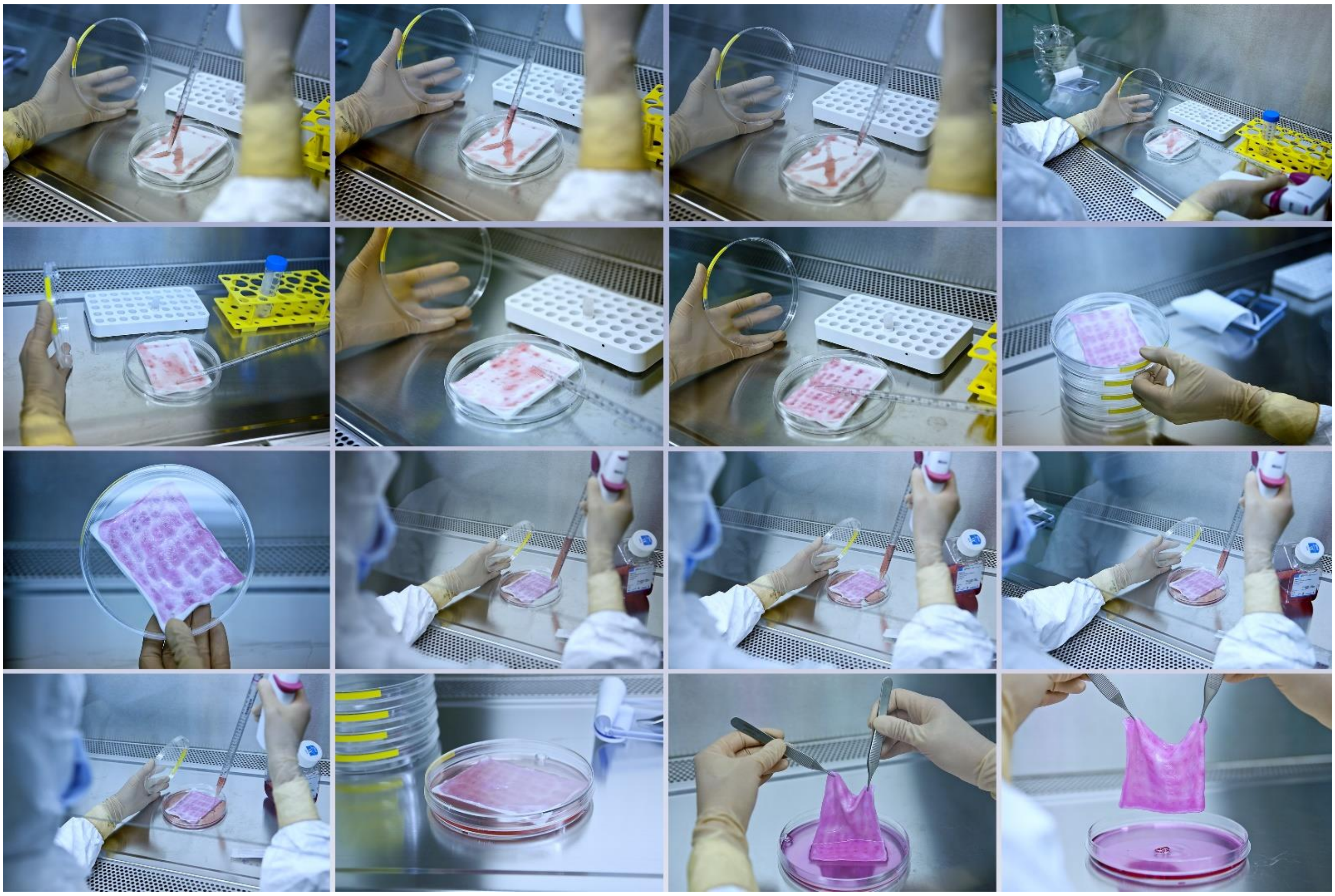

Figure S16 (*cont.*). Illustrated stepwise overview of PBB construct preparation (*part 2/2*). Following consistent and homogeneous repartition of the therapeutic cell suspension on the scaffold, the PBB lot is appropriately incubated for 24–48 hours to favour cellular colonization of the scaffold. The PBBs are then ready for conditioning and for delivery to the clinic. Following incubation, always hold and gently manipulate the PBB constructs in a vertical position, using two sterile forceps to hold both upper corners of the constructs. For removal of the constructs from the primary packaging and application on the patient, use a rolling motion to progressively lift or deposit the constructs (i.e., do not slide or reposition the constructs once applied), starting with the upper or lower edge of the constructs, respectively.

© Swiss progenitor cell transplantation program

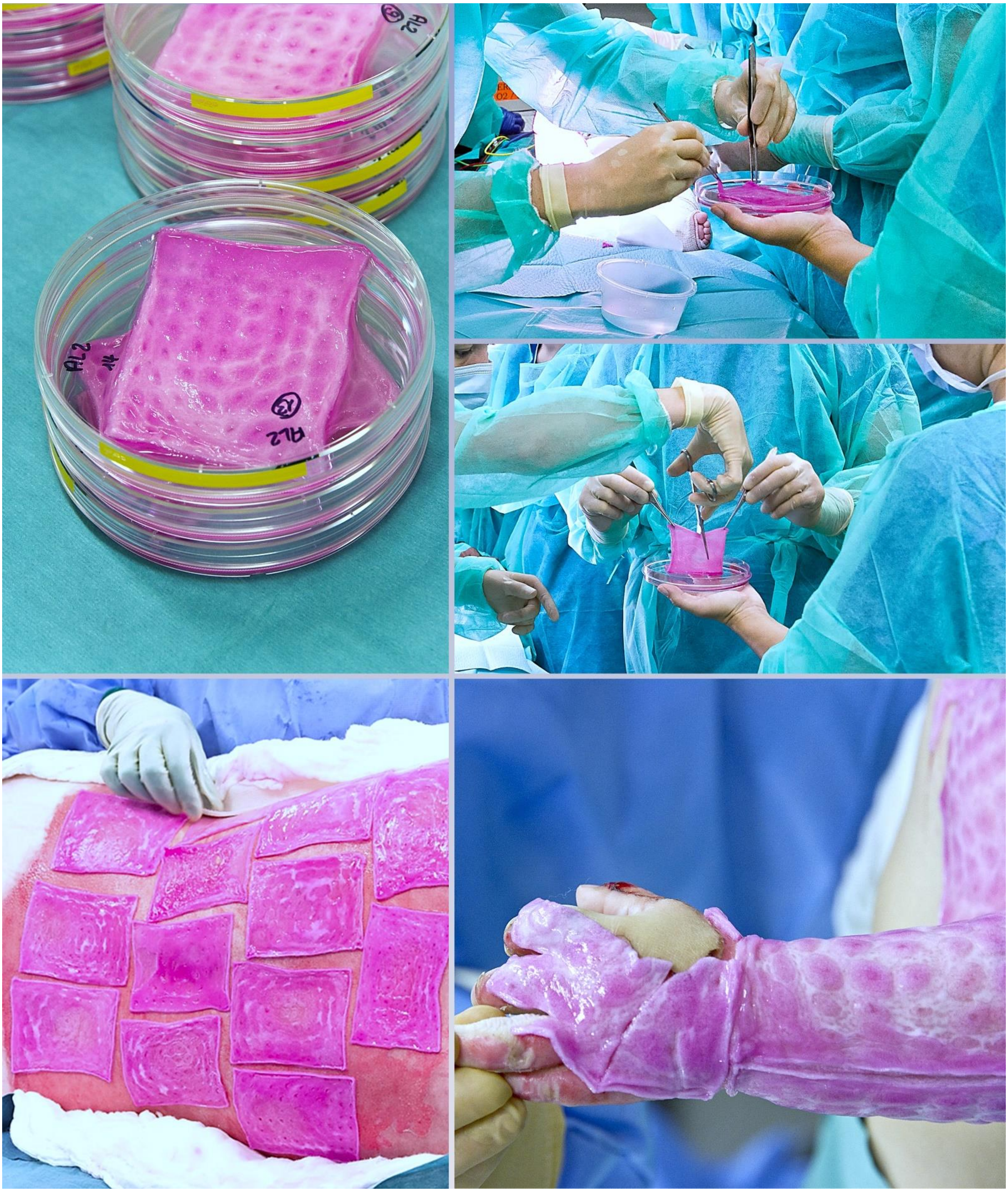

Figure S17. Illustrations of PBB lot reception and use in the operating theatre. The illustration shows the aspect of a PBB lot as delivered to the clinic in the primary packaging (*upper left quadrant*). Illustrations on how to hold and cut the PBB constructs are provided (*upper right quadrant*). Illustrations on how to position the PBB constructs on large planar wounds or on small and topographically complex wounds are respectively provided (*both lower quadrants*).

© Swiss progenitor cell transplantation program
