# Supplementary material for: Retrospectives on Three Decades of Safe Clinical Experience with Allogeneic Dermal Progenitor Fibroblasts: High Versatility in Topical Cytotherapeutic Care
Source: Pharmaceutics. 2023 Jan 4;15(1):184. doi: 10.3390/pharmaceutics15010184 (PMC9866885; doi:10.3390/pharmaceutics15010184)
Supplement: Supplementary file 1 [file pharmaceutics-15-00184-s001.zip › Supplementary Figures.pdf]

Supplementary Figures

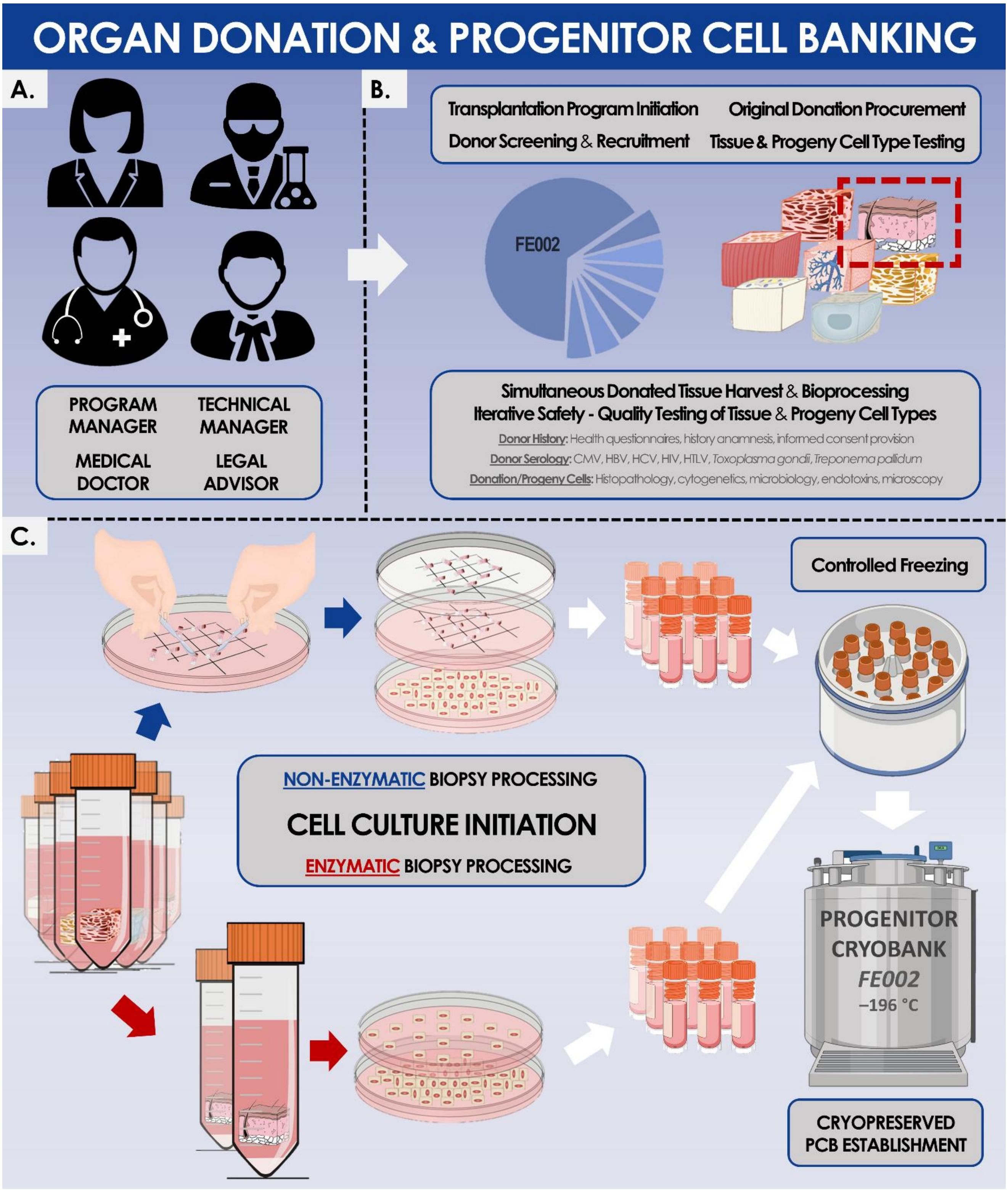

Figure S1. Organizational and technical overview of the Swiss progenitor cell transplantation program for primary progenitor cell sourcing, manufacturing, and clinical cytotherapeutic use in regenerative medicine. (**A**) Schematic representation of professional stakeholders who are required at minimum for the devising and operation of a progenitor cell transplantation program. (**B**) Essential methodological elements for the procurement, testing, and simultaneous bioprocessing of donated tissue types. (**C**) Critical steps for the culture initiation of primary progenitor cell types (e.g., FE002-SK2 cells), parallelly using enzymatic and mechanical tissue processing methodologies. The resulting cultures are harvested and used to constitute original cryopreserved PCB lots. CMV, cytomegalovirus; HBV, hepatitis B virus; HCV, hepatitis C virus; HTLV, human T-cell lymphotropic virus; PCB, parental cell bank.

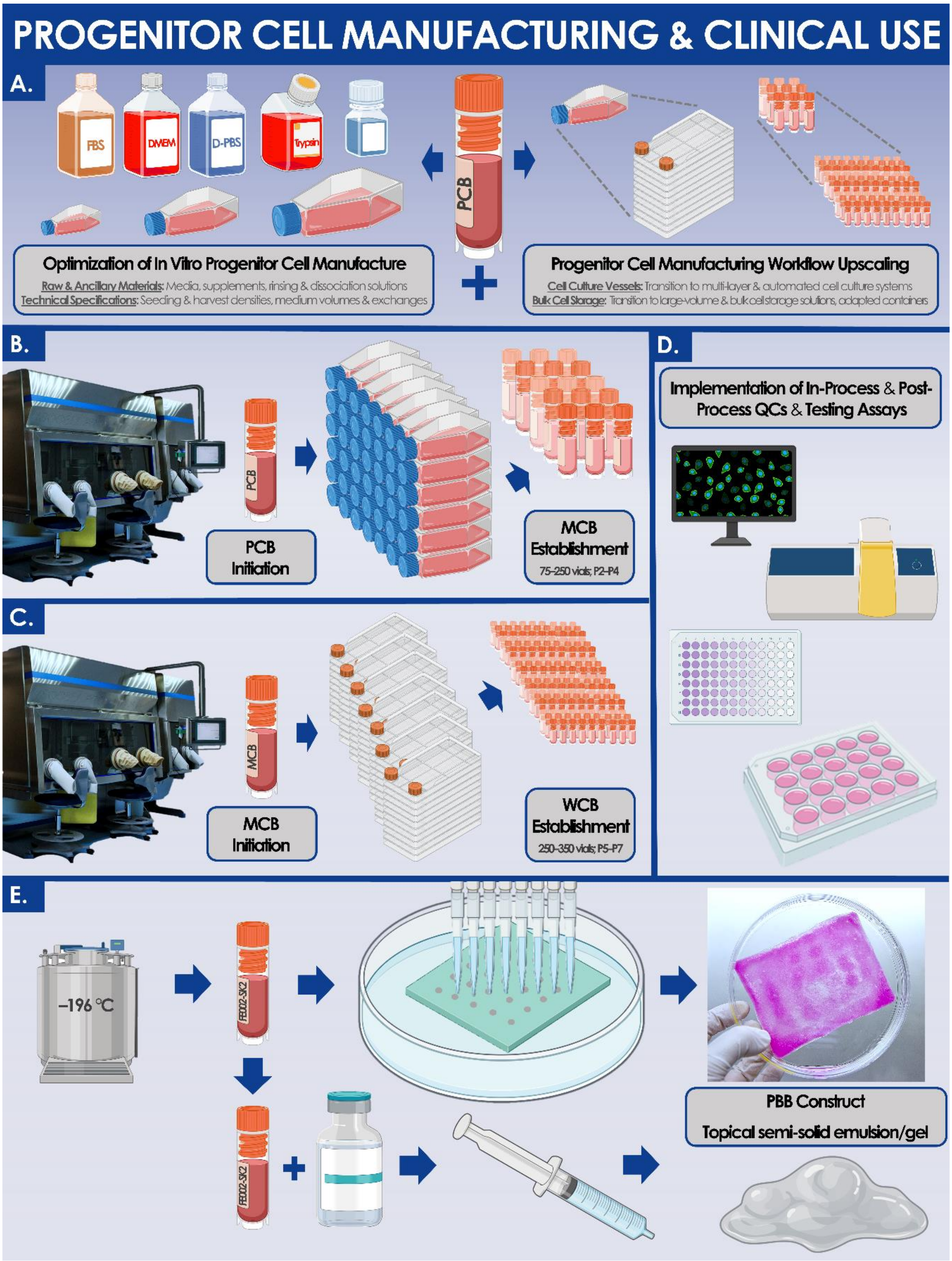

Figure S2. Technical workflows for clinical-grade progenitor cell manufacture and formulation into topical cytotherapeutic products. (**A**) Biphasic technical optimization performed on each new primary progenitor cell type, using a pilot working cell bank. The in vitro culture conditions and the materials to use for manufacture are firstly determined, followed by manufacturing workflow upscaling and adaptation to industrial GMP processes. (**B**) PCB materials are used for GMP culture expansion and constitution of an MCB. (**C**) MCB materials are used for GMP culture expansion and constitution of a WCB. (**D**) Appropriate monitoring and testing quality controls are implemented for the release and qualification of the produced cell lots. (**E**) WCB materials are eventually used as APIs for topical cytotherapeutic product extemporaneous reconstitution. API, active pharmaceutical ingredient; DMEM, Dulbecco's modified Eagle medium; D-PBS, Dulbecco's phosphate-buffered saline; FBS, fetal bovine serum; GMP, good manufacturing practices; MCB, master cell bank; P, in vitro passage level; PBB, progenitor biological bandage; PCB, parental cell bank; QC, quality control; WCB, working cell bank.

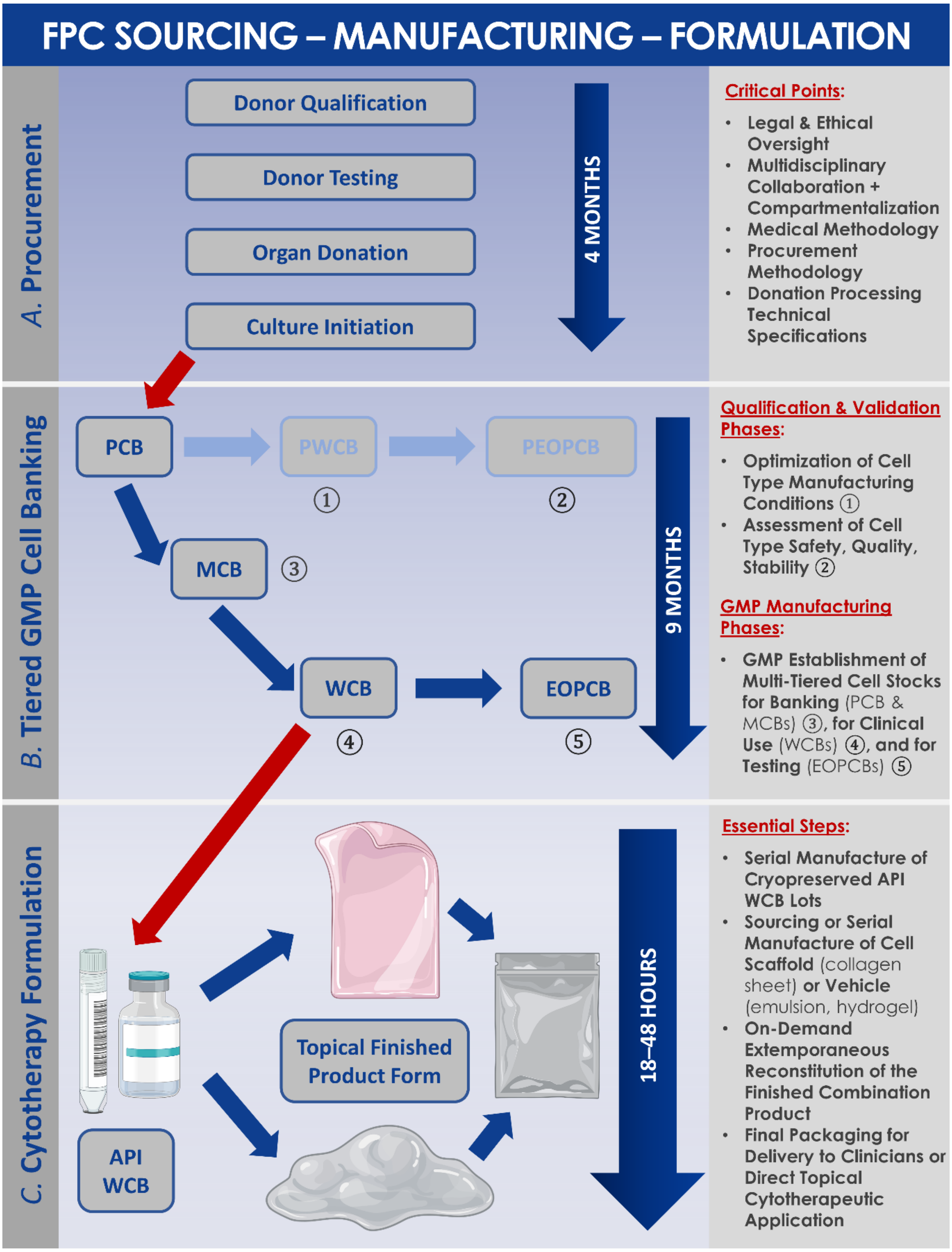

Figure S3. Illustrated stepwise workflow succinctly presenting the key phases of fibroblastic progenitor cell sourcing, GMP cell manufacture, and finished cytotherapeutic product formulation. (**A**) Procurement of the organ donation is only necessary once for the derivation of several billion WCB API doses. Extreme importance is set on methodological aspects of the original procurement. (**B**) Following qualification and validation phases aiming to ensure that the primary cell type is of sufficient quality for application in human regenerative medicine, serial GMP manufacturing campaigns may be carried out. (**C**) Starting with the cryopreserved form of the cellular API, the finished product form (e.g., PBB constructs) may be extemporaneously reconstituted for topical cytotherapeutic clinical care. API, active pharmaceutical ingredient; EOPCB, end of production cell bank; FPC, fibroblastic progenitor cells; MCB, master cell bank; PBB, progenitor biological bandage; PCB, parental cell bank; PEOPCB, pilot end of production cell bank; PWCB, pilot working cell bank; WCB, working cell bank.

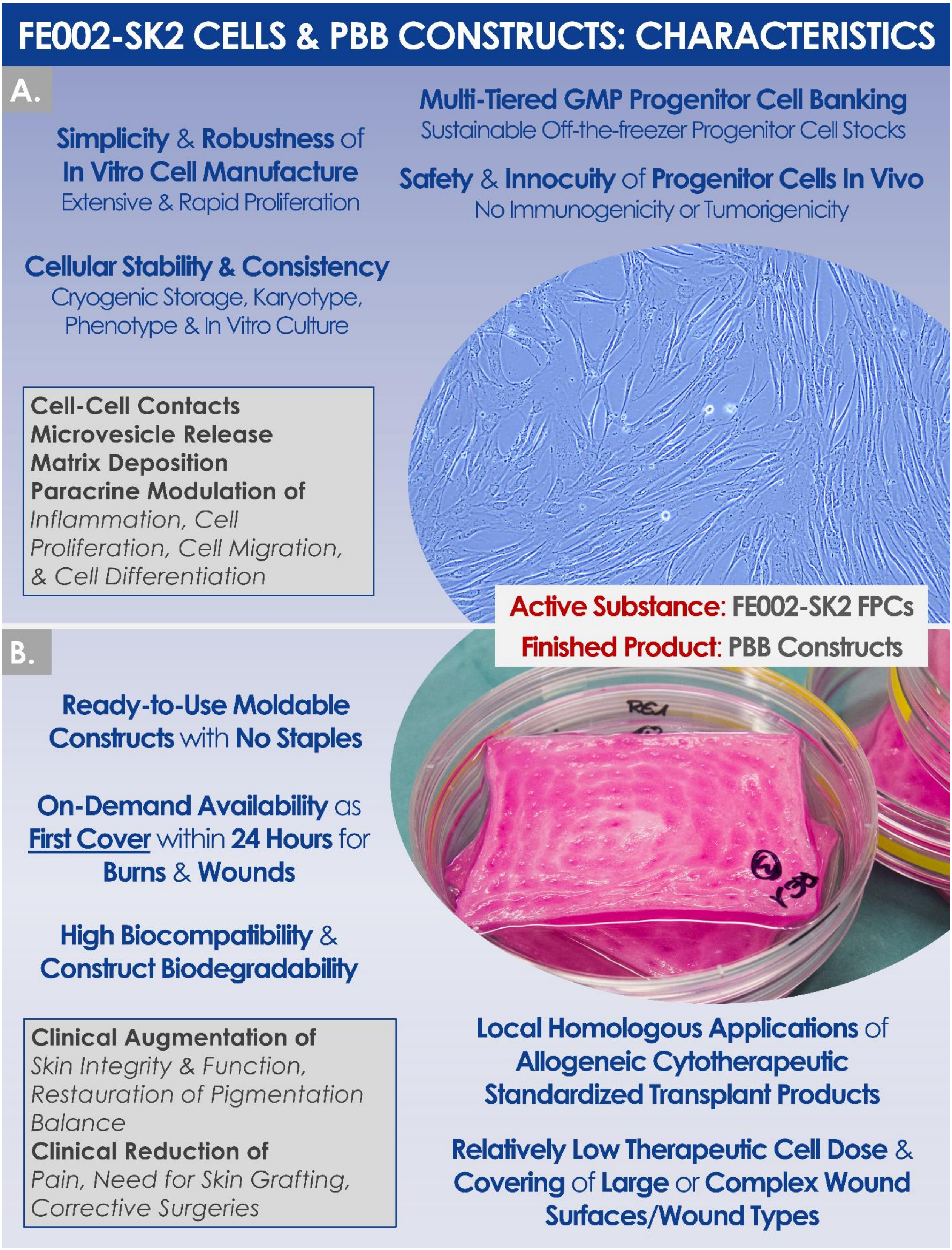

Figure S4. Illustrated overview of the main characteristics and advantages of using cultured progenitor cells (e.g., FE002-SK2 cell type) as an active substance for allogeneic standardized transplant products (e.g., PBB constructs). (**A**) Main characteristics of the cellular active substance of interest, with demonstrated or postulated effects (i.e., shown in the grey quadrant). (**B**) Main characteristics of the PBB topical cytotherapeutic early wound dressing solution for clinical application in the Lausanne burn center, with demonstrated effects (i.e., shown in the grey quadrant). FPC, fibroblastic progenitor cells; GMP, good manufacturing practices; PBB, progenitor biological bandages.

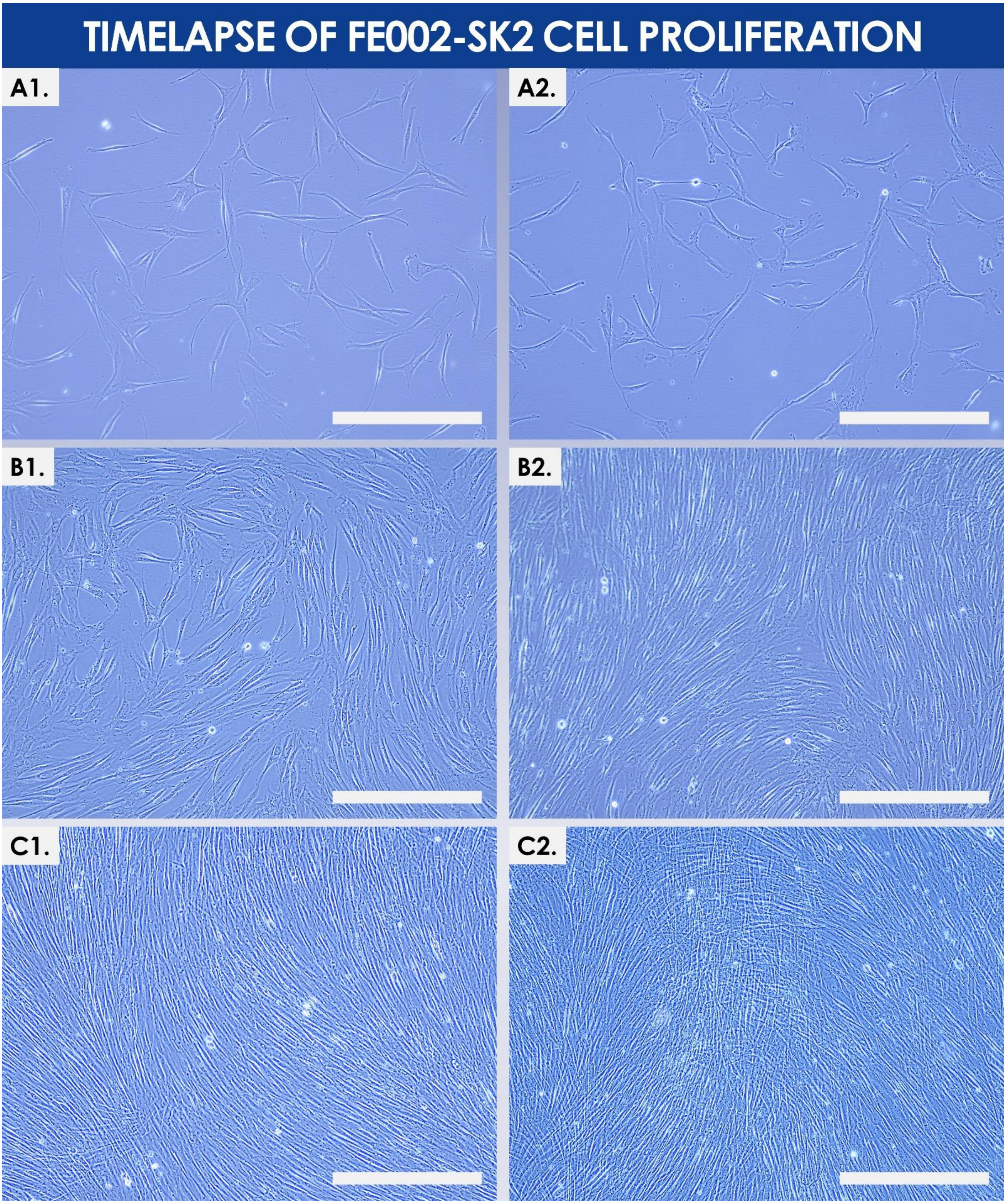

Figure S5. Evolutive cellular morphology of proliferating FE002-SK2 primary progenitor cells in monolayer culture at passage level 6. The photographic records of in vitro culture vessels were constituted following image acquisition using phase contrast microscopy. At each timepoint, cell monolayers from two distinct culture vessels were imaged. (**A**) Proliferating cells after 48 h of culture. (**B**) Proliferating cells after 10 days of culture. (**C**) Proliferating cells after 14 days of culture. The FE002-SK2 cell type was deposited in 2012 in the ECACC, N°12070301-FE002-SK2 and in the FIRDI, N°BCRC 960460. Scale bars = 100 µm. h, hours.

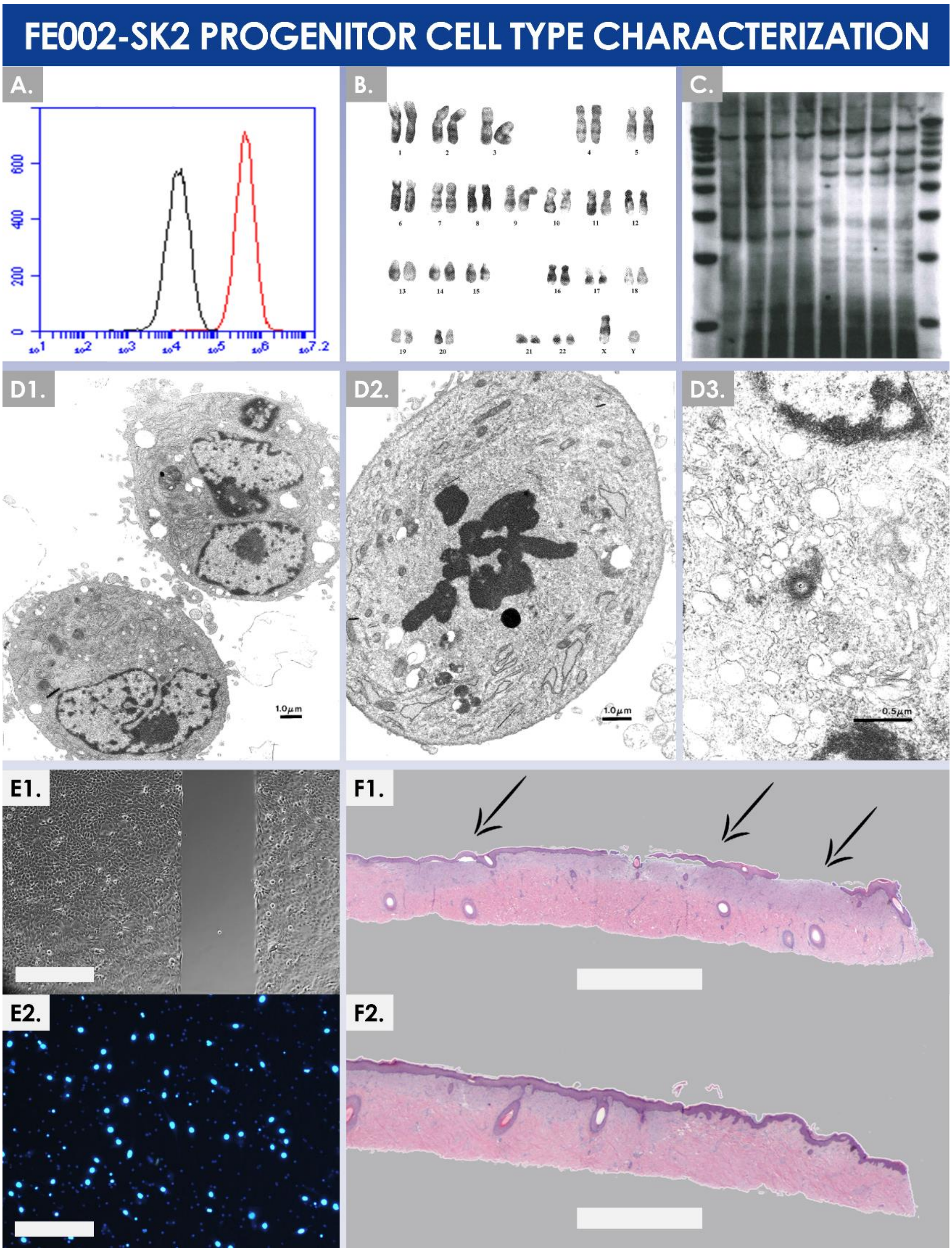

Figure S6. Illustrated overview of various selected examples for descriptive and qualitative assays, in vitro functional assays, and an in vivo safety study performed for the characterization of FE002-SK2 primary progenitor cells. (**A**) FACS data plot showing positive expression of the CD44 cell surface marker. (**B**) Karyotyping analysis for the investigation of cell type genetic stability. (**C**) DNA fingerprinting analysis result of the FE002-SK2 cell type. (**D**) Representative TEM micrographs of FE002-SK2 cells. Scale bars = 0.5 µm or 1.0 µm. (**E**) In vitro assays for the demonstration of primary keratinocyte migration stimulation potential by FE002-SK2 cells or derivatives, in a scratch-assay setup (**E1**) and in a Transwell® cell culture setup (**E2**), respectively. (**F**) Histological results of a GLP porcine study of the effects of the cells on an excision wound model. The slide presented in **F1** corresponds to the control group, displaying epidermal detachment (i.e., as indicated by arrows), while the slide presented in **F2** corresponds to a cell treatment group, displaying no epidermal detachment. Scale bars = 500 µm. CD, cluster of differentiation; DNA, deoxyribonucleic acid; FACS, fluorescence-activated cell sorting; GLP, good laboratory practices; TEM transmission electron microscopy. (**A**–**D**) Modified and adapted with permission from Laurent et al., 2021 [38]. (**E**–**F**) Modified and adapted with permission from Laurent et al., 2020 [5].

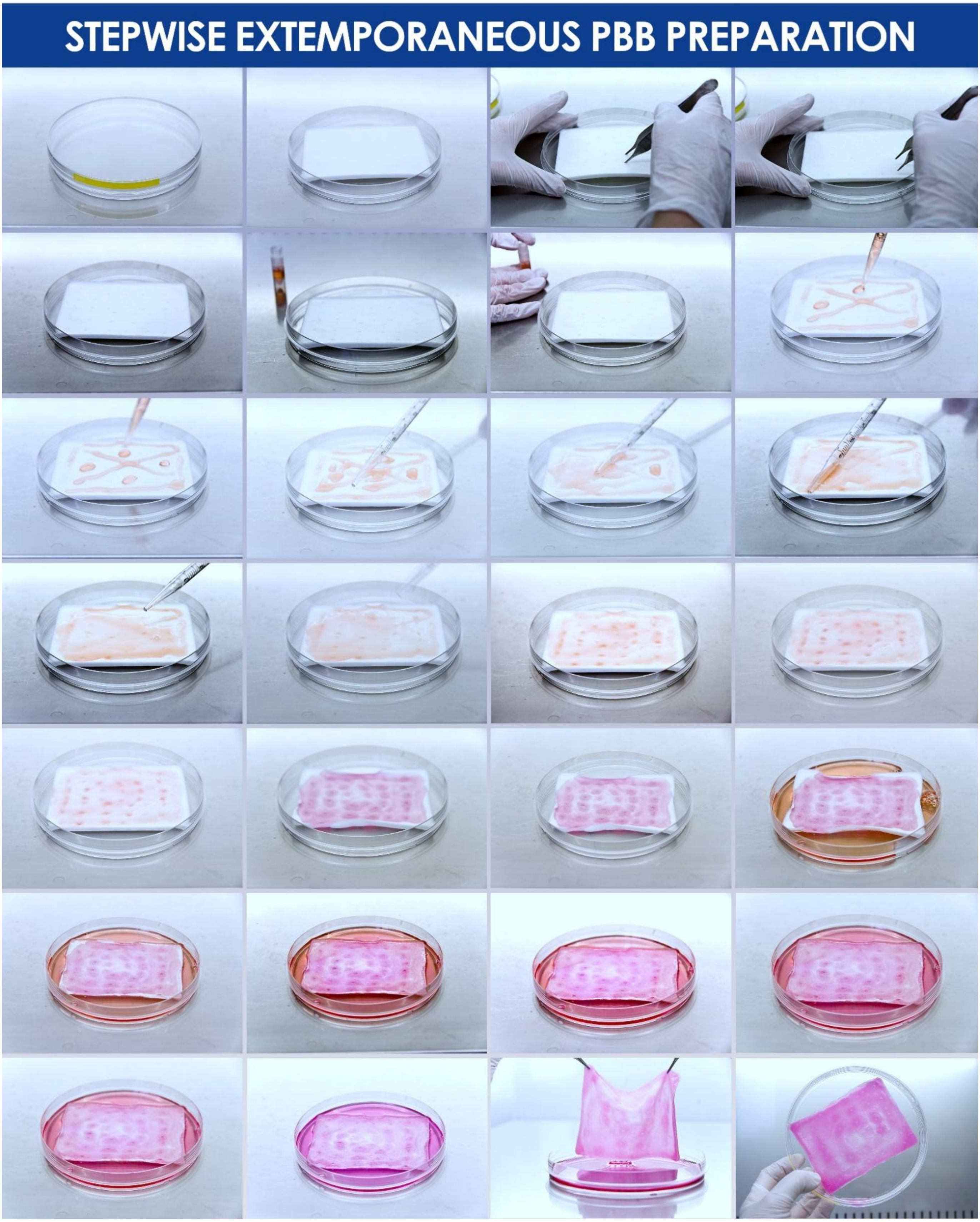

Figure S7. Illustrative photographic timelapse of the extemporaneous preparation of a PBB cytotherapeutic product unit. Following initial mechanical conditioning of the collagen scaffold, the primary progenitor cell suspension (e.g., FE002-SK2 cells) is homogeneously seeded across the available scaffold surface. The construct is then incubated with appropriate cell culture medium for 18–24 hours to favor cell adherence and scaffold colonization by the cells. Finally, the construct is conditioned in transport medium for delivery to the operating theatre. PBB, progenitor biological bandage.

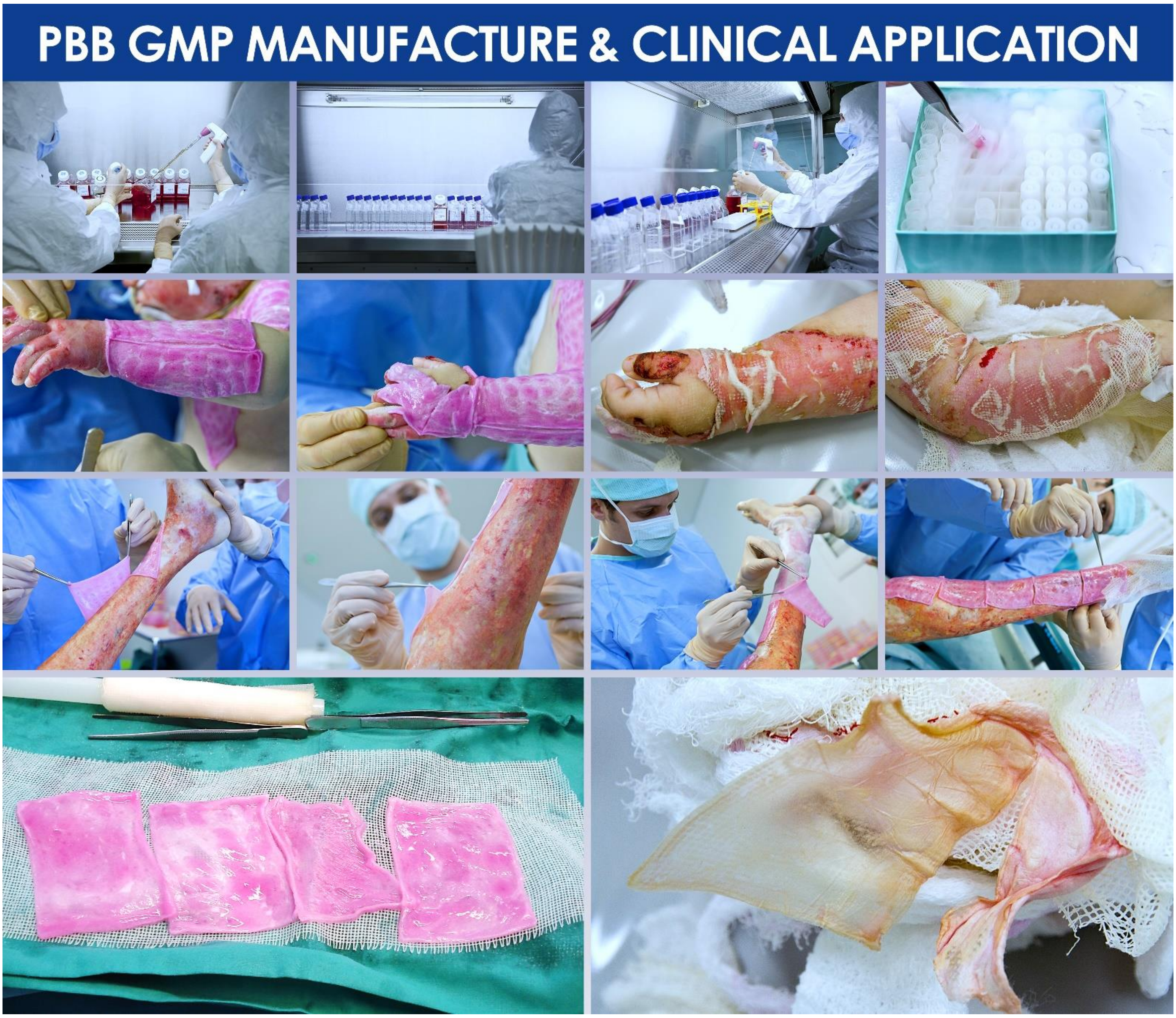

Figure S8. Illustrative photographic overview of progenitor cytotherapeutic API in vitro processing and clinical PBB administration in the Lausanne burn center (CHUV, Lausanne, Switzerland). Following aseptic processing of the cellular API, an off-the-freezer cryopreserved cell stock is constituted and is made available for clinician prescription. PBBs are delivered on-demand in the operating theatre and are applied on debrided burn wounds during the bandage application and exchange procedures. PBBs may be molded or adapted in size to fit any anatomical location on the patient and are maintained in place by gauze and standard bandaging, without the need for staples or adhesives. During subsequent bandage exchange procedures, the old PBBs are usually washed off as a part of standard wound care. API, active pharmaceutical ingredient; CHUV, centre hospitalier universitaire vaudois; GMP, good manufacturing practices; PBB, progenitor biological bandage.

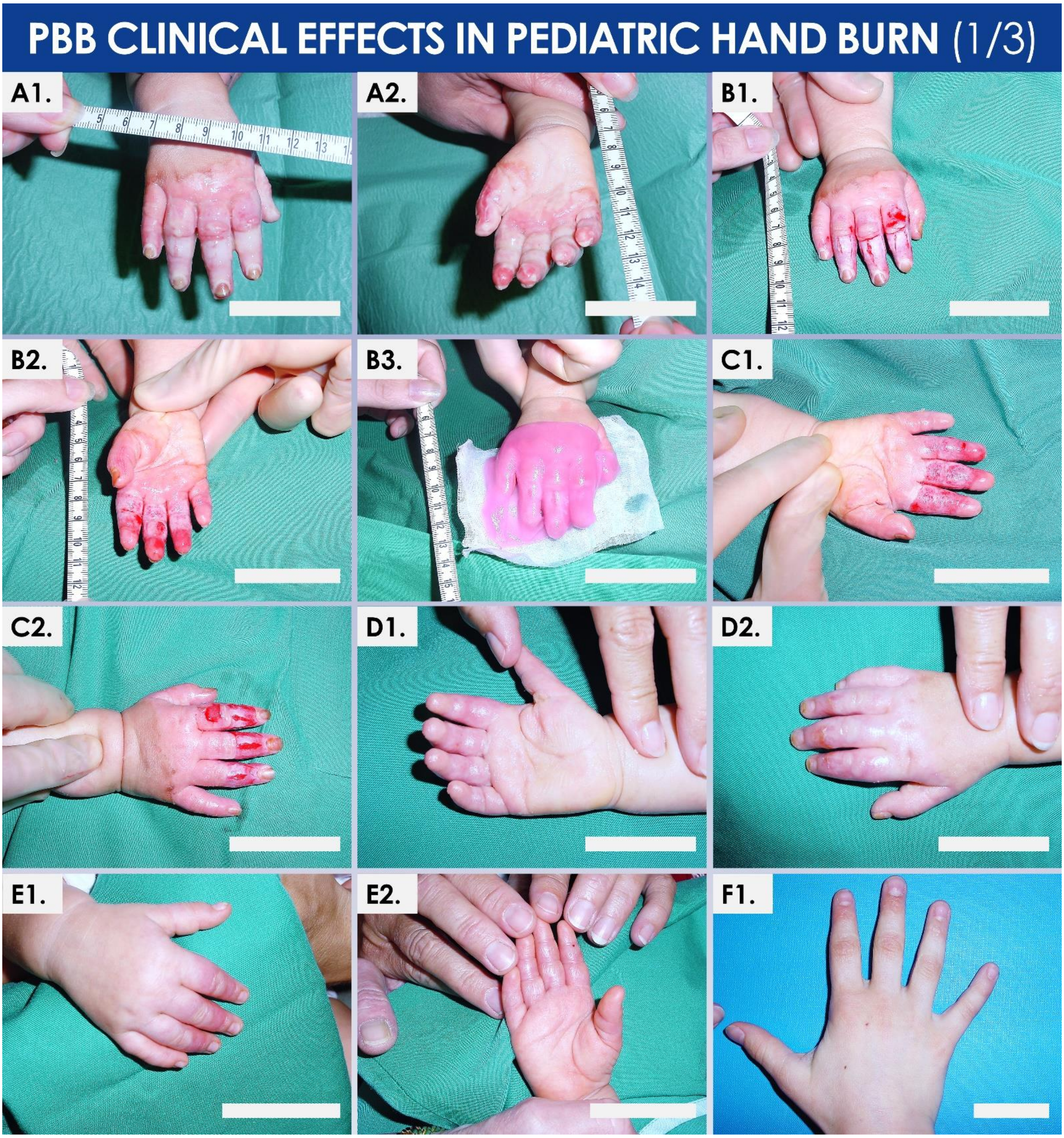

Figure S9. Illustrative examples of PBB clinical therapeutic results in burn wound cytotherapeutic care. The depicted clinical case corresponded to a 14-month-old female patient presenting second-degree and third-degree pediatric burn wounds (i.e., wound surface of 28.8 cm^2) of the hand caused by scalding liquid. Treatment was introduced 8 days after the trauma. A total of five PBB constructs were applied every 2–3 days and the wounds had completely closed after 15 days. (**A**) Cutaneous lesions before wound debridement. (**B**) Cutaneous lesions after wound debridement and PBB application. (**C**) Cutaneous lesions after the initial PBB treatment. (**D**) Cutaneous lesions after the end of PBB treatments, 23 days after the initial debridement. (**E**) Follow-up, 43 days after the initial debridement. (**F**) Follow-up, 7 years after the treatment. Scale bars = 4 cm. Modified and adapted with permission from Laurent et al., 2020 [18].

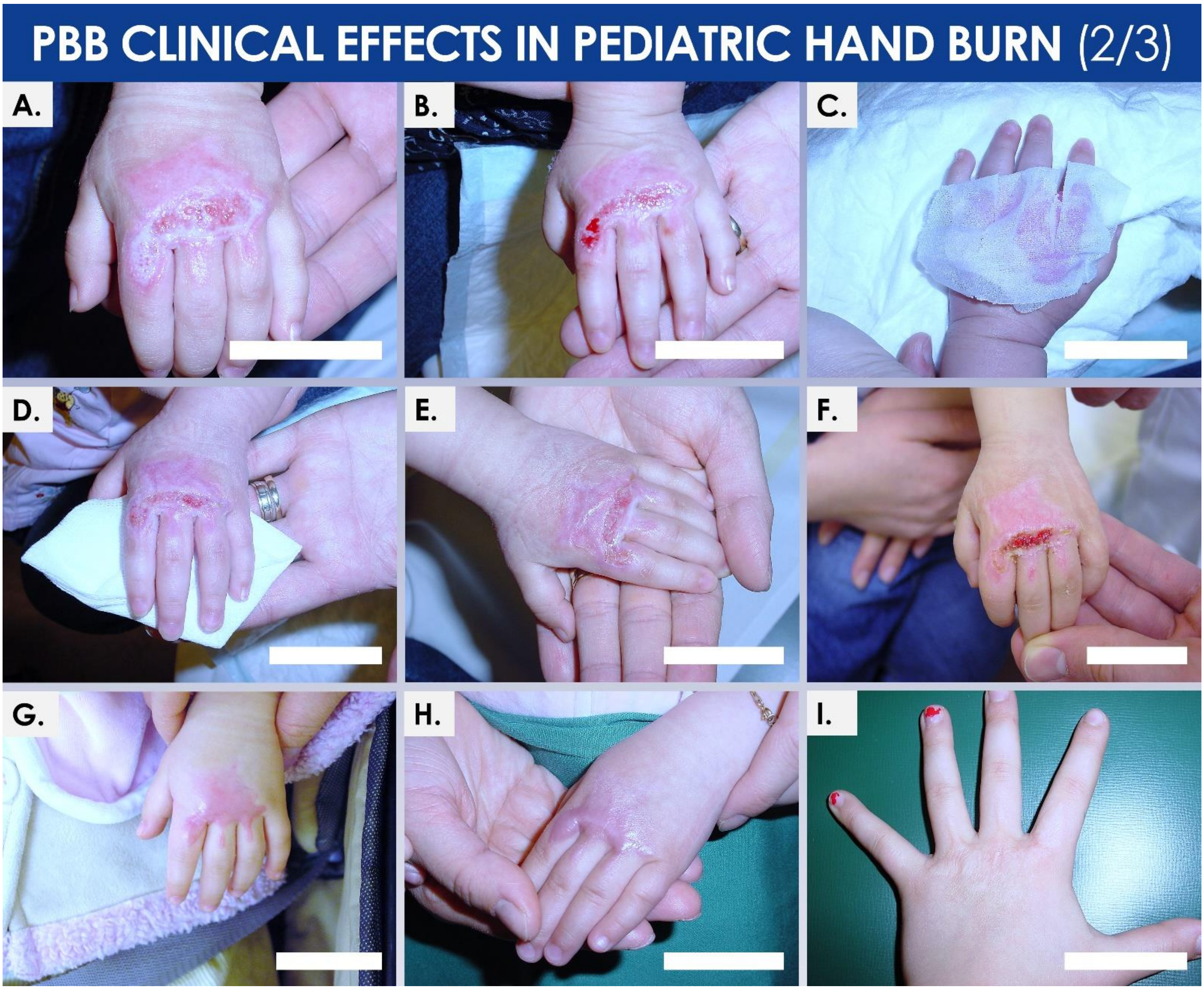

Figure S10. Illustrative examples of PBB clinical therapeutic results in burn wound cytotherapeutic care. The depicted clinical case corresponded to a 17-month-old female patient presenting second-degree and third-degree pediatric burn wounds (i.e., wound surface of 12.2 cm^2) of the hand caused by an iron. Treatment was introduced 5 days after the trauma. A total of three PBB constructs were applied every 2–3 days and the wounds had completely closed after 7 days. (**A**) Cutaneous lesions before wound debridement and application of the first construct (i.e., day 1). (**B**) Cutaneous lesions after wound debridement and before application of the second construct (i.e., day 3). (**C**) Cutaneous lesions after PBB application (i.e., second construct, day 3). (**D**) Cutaneous lesions before application of the third construct (i.e., day 5). (**E**) Cutaneous lesions after application of the third construct (i.e., day 7). (**F**) Cutaneous lesions at day 12. (**G**) Cutaneous lesions at day 60. (**H**) Cutaneous lesions at day 165. Some hypertrophic scarring required silicone application. (**I**) Follow-up, 7 years after the treatment. Scale bars = 4 cm. PBB, progenitor biological bandage.

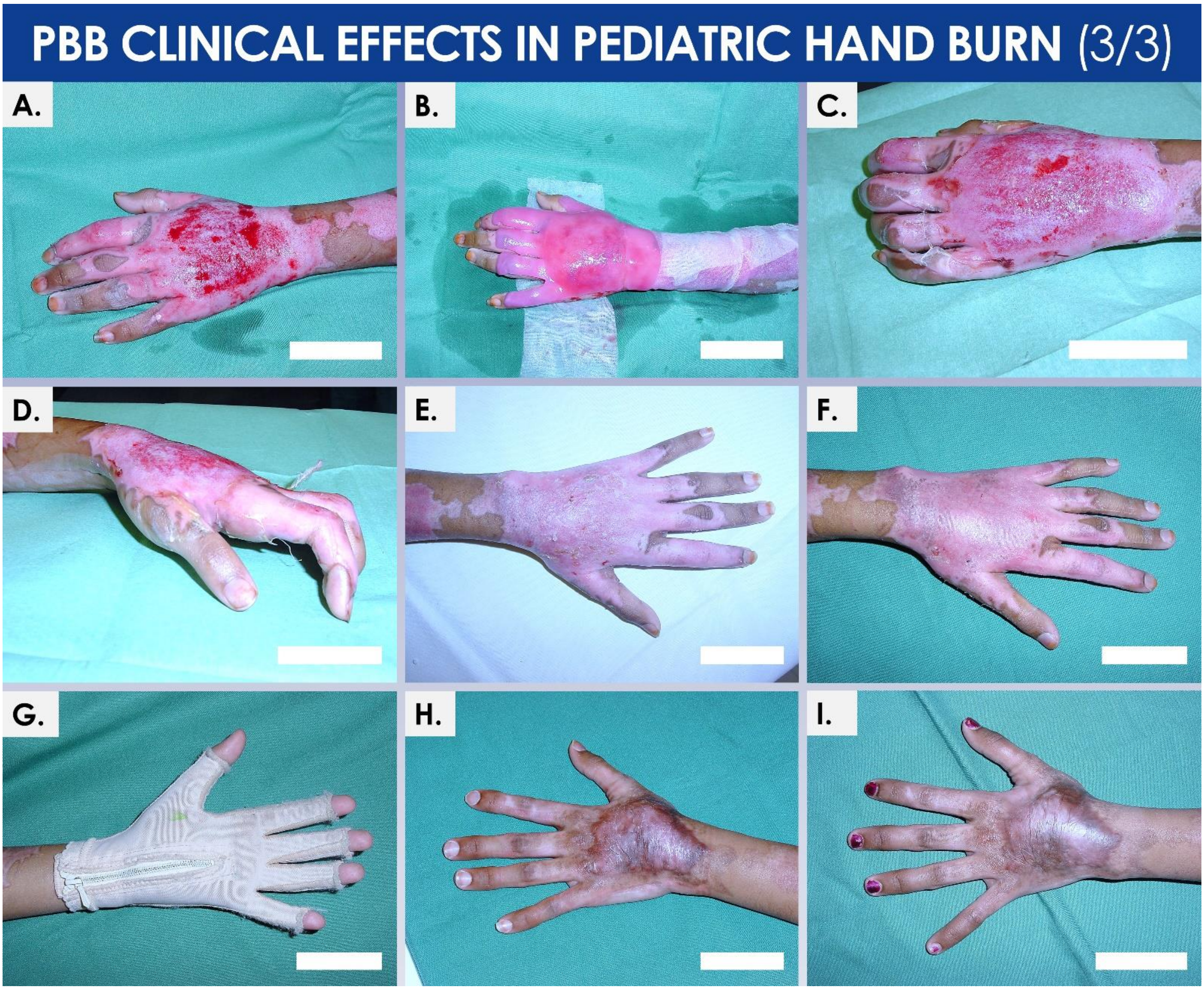

Figure S11. Illustrative examples of PBB clinical therapeutic results in burn wound cytotherapeutic care. The depicted clinical case corresponded to a 9-year-old female patient presenting second-degree and third-degree pediatric burn wounds (i.e., wound surface of 130.9 cm^2) of the hand caused by hot oil. Treatment was introduced 10 days after the trauma. A total of three PBB constructs were applied successively, and the wounds had completely closed after 17 days. (**A**) Cutaneous lesions before application of the first construct (i.e., day 1). (**B**) Cutaneous lesions after application of the first construct (i.e., day 1). (**C**–**D**) Cutaneous lesions before application of the second construct (i.e., day 3). (**E**) Cutaneous lesions after application of the third construct (i.e., day 17). The third construct had been applied on day 8. (**F**) Follow-up of the wounds after standard care (i.e., day 25). (**G**) Fitting of compressive garment (i.e., day 25). (**H**) Follow-up of the wounds, 6 months after the treatment. (**I**) Follow-up of the wounds, 7 months after the treatment. Scale bars = 4 cm. PBB, progenitor biological bandage.

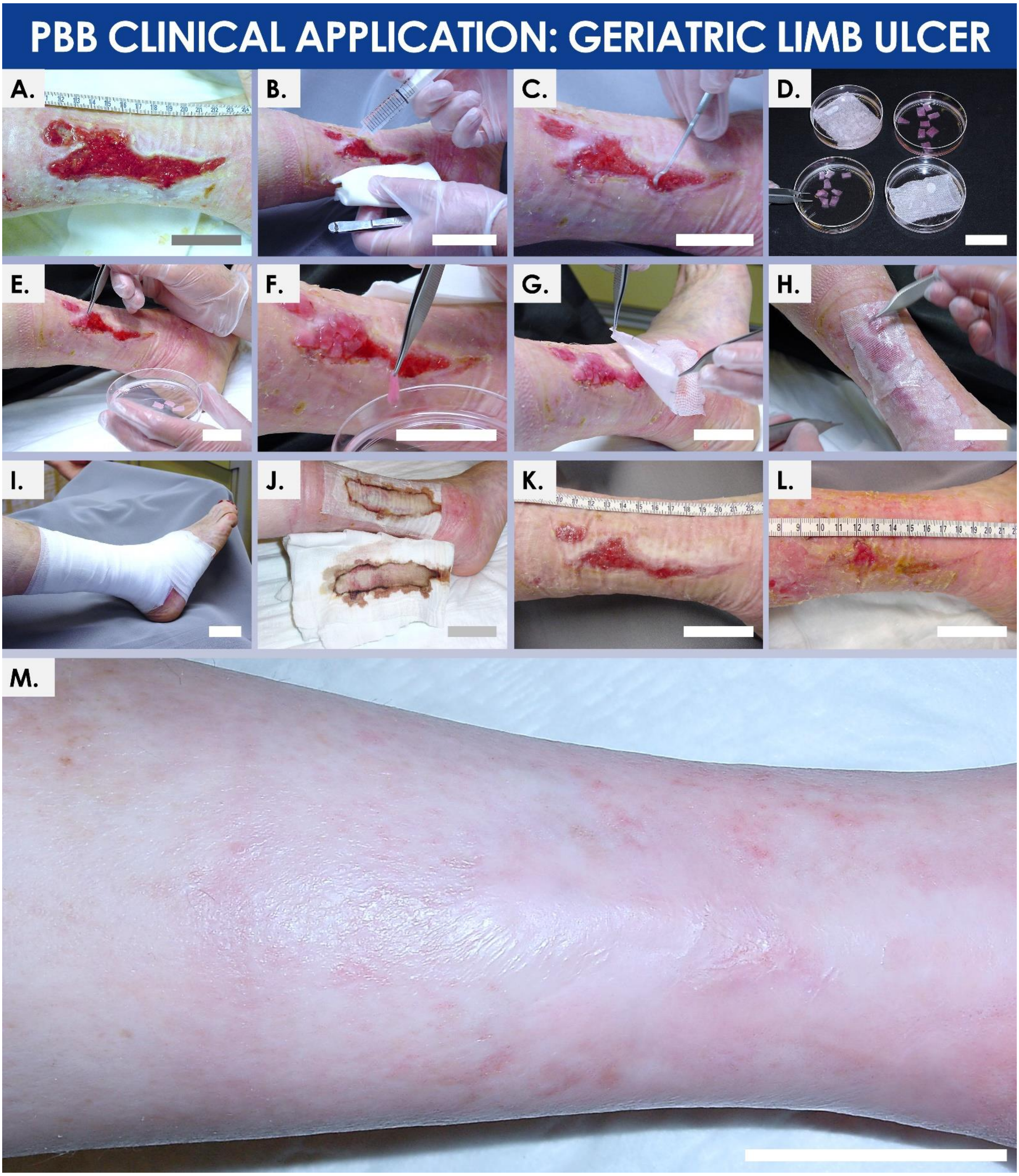

Figure S12. Illustrative examples of PBB clinical therapeutic application in refractory post-thrombotic ulcer lesions in geriatric patients. The lesions were treated weekly with PBB subunits, which were adapted to closely fit the wounds. (**A**) Initial status of the wound at the time of treatment instauration (i.e., day 1). (**B**) Rinsing and humidification of the wound prior to debridement. (**C**) Debridement of the wound. (**D**) Preparation of PBB construct subunits to fit the dimensions of the wound bed. (**E**–**H**) Stepwise application of the construct subunits under a covering bandage. (**I**) Secondary bandages covering the treated wound. (**J**) Removal of the bandages during the bandage exchange procedure. (**K**) Status of the wound 35 days after treatment instauration. (**L**) Status of the wound 70 days after treatment instauration. (**M**) Follow-up of the patient 1 year after treatment instauration. Scale bars = 4 cm. PBB, progenitor biological bandage.

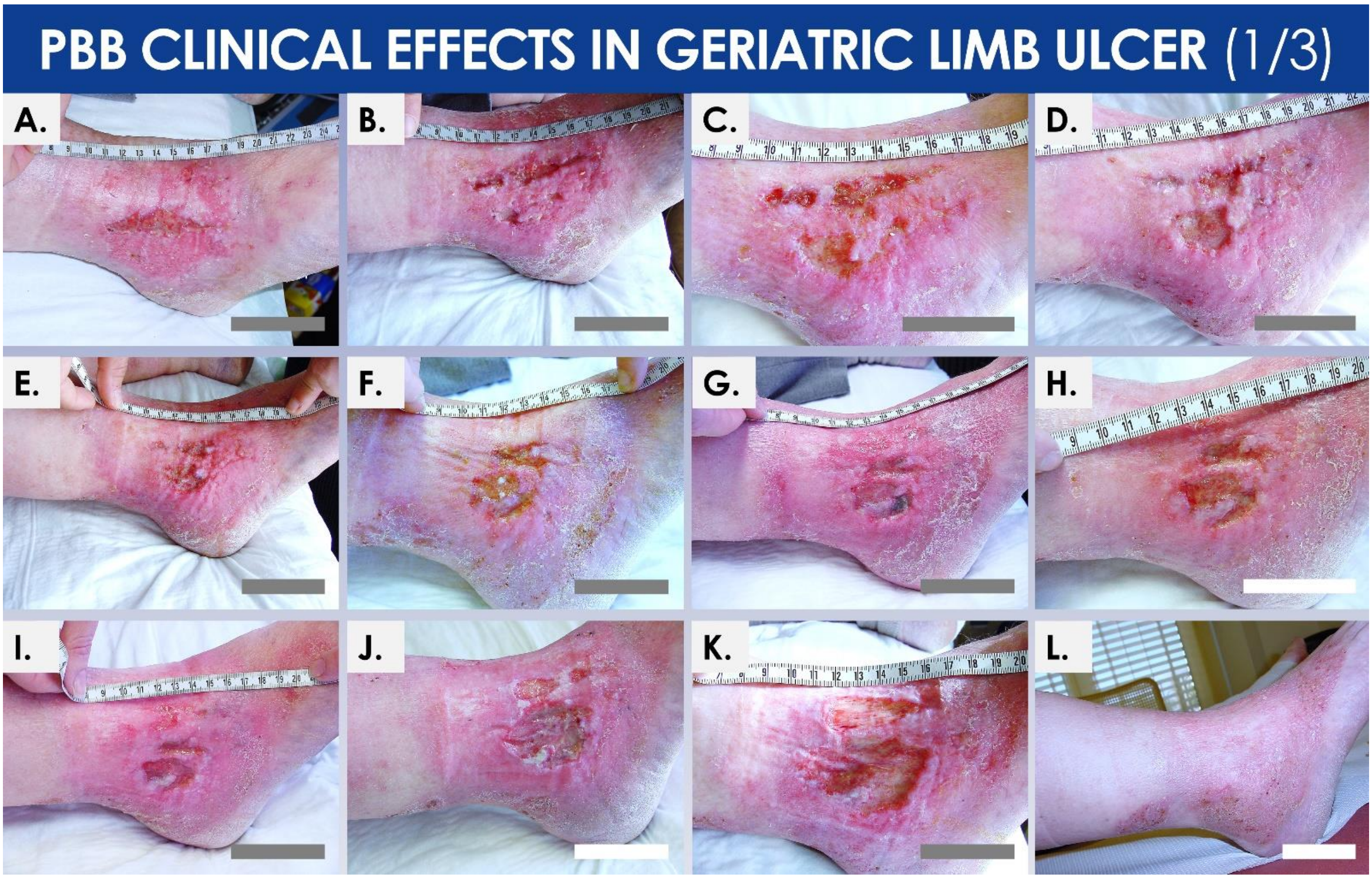

Figure S13. Illustrative examples of PBB clinical therapeutic results in geriatric lower-limb cutaneous ulcer cytotherapeutic care. The depicted clinical case corresponded to a 37-year-old female patient suffering for more than ten years from a severe post-traumatic post-thrombotic syndrome, with atrophie blanche. Topical weekly treatment with PBBs resulted in rapid healing and relieving of pain, despite intermittent and repetitive inflammatory outbreaks. (**A**) Initiation of the treatment at M_0. (**B**) Wound status at M_1. (**C**) Wound status at M_2. (**D**) Wound status at M_3. (**E**) Wound status at M_4. (**F**–**G**) Wound status at M_5. (**H**–**I**) Wound status at M_6. (**J**–**K**) Wound status at M_7. (**L**) Wound status at M_{13}. No pain was felt by the patient during the entire course of the treatment. At the end of the treatment, the wound was completely closed. Scale bars = 4 cm. M_X, month X; PBB, progenitor biological bandage.

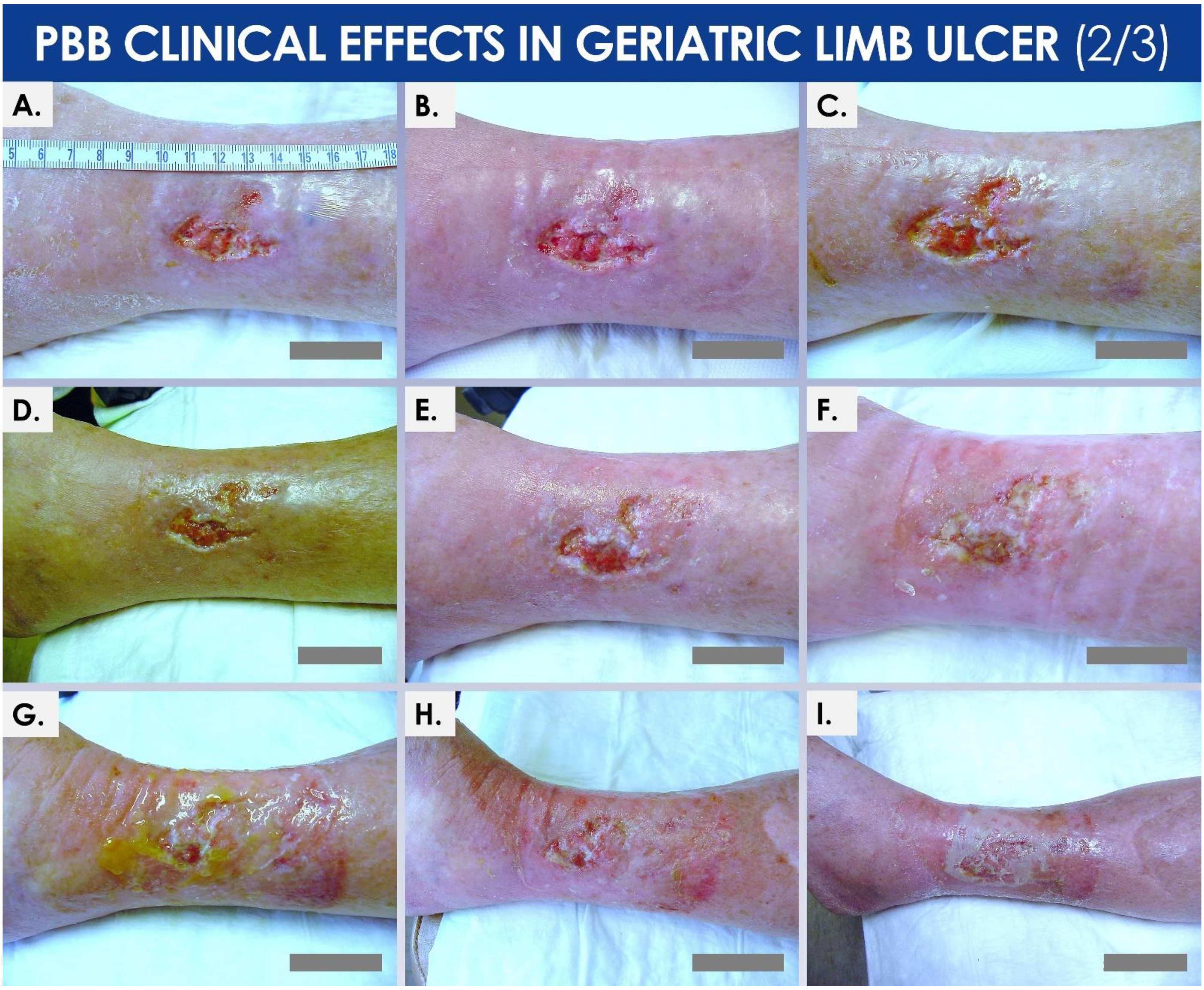

Figure S14. Illustrative examples of PBB clinical therapeutic results in geriatric lower-limb cutaneous ulcer cytotherapeutic care. The depicted clinical case corresponded to an 87-year-old female patient suffering from leg ulcers due to arterial insufficiency. Topical weekly treatment with PBBs resulted in rapid healing and relieving of pain, allowing for a reduction in the intake of analgesic drugs during the course of the treatment. (**A**) Initiation of the treatment at week 0. (**B**) Wound status at week 1. (**C**) Wound status at week 2. (**D**) Wound status at week 4. (**E**) Wound status at week 5. (**F**) Wound status at week 7. (**G**) Wound status at week 8. (**H**–**I**) Wound status at week 9. Scale bars = 4 cm. PBB, progenitor biological bandage.

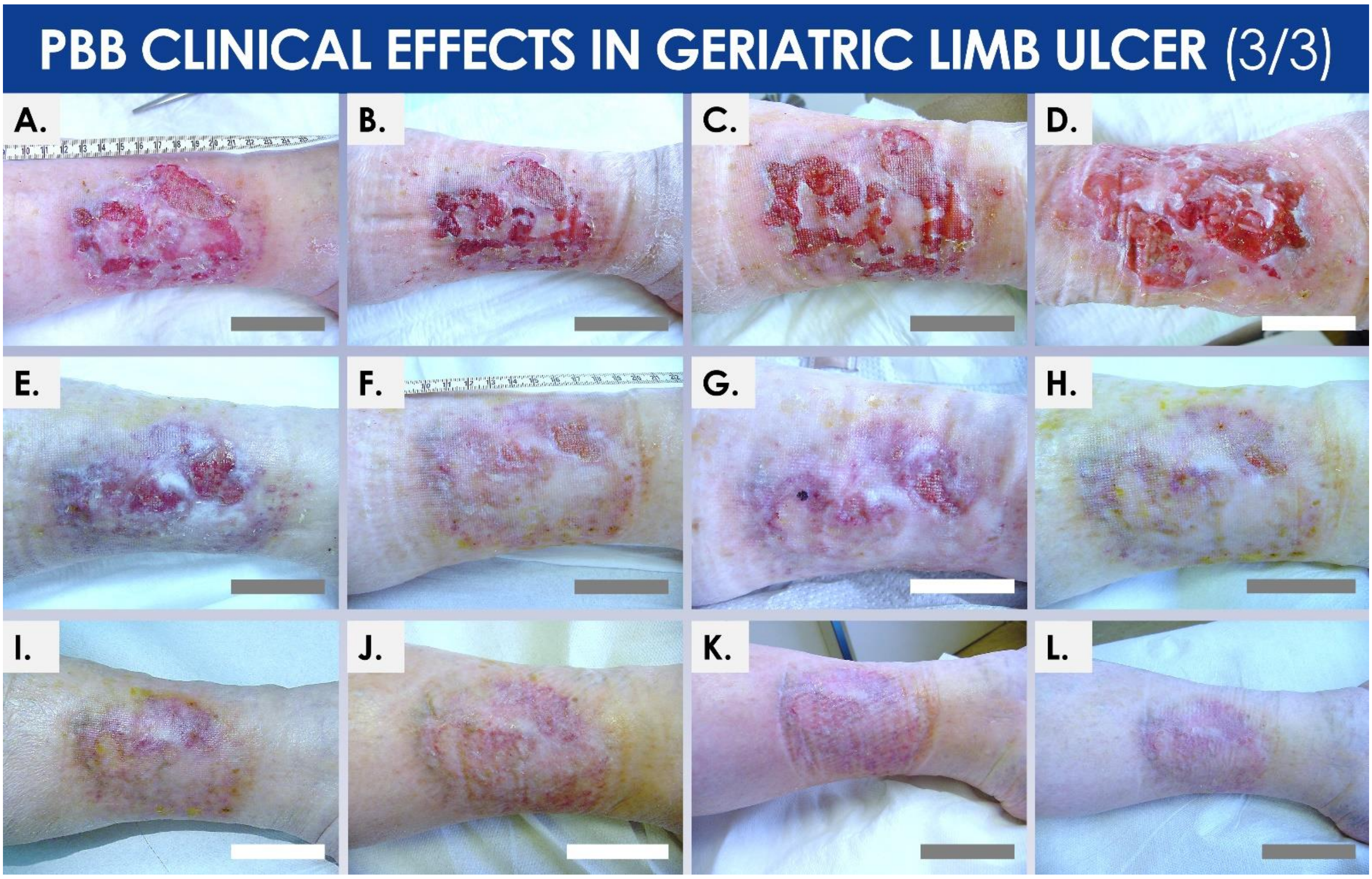

Figure S15. Illustrative examples of PBB clinical therapeutic results in geriatric lower-limb cutaneous ulcer cytotherapeutic care. The depicted clinical case corresponded to a 72-year-old female patient suffering from leg ulcers due to arterial insufficiency. Topical weekly treatment with PBBs resulted in rapid healing and relieving of pain. (**A**) Initiation of the treatment at week 0. (**B**) Wound status at week 1. (**C**) Wound status at week 2. (**D**) Wound status at week 3. (**E**) Wound status at week 4. (**F**) Wound status at week 5. (**G**) Wound status at week 6. (**H**) Wound status at week 7. (**I**) Wound status at week 9. (**J**) Wound status at week 12. (**K**) Wound status at week 13. (**L**) Wound status at week 17. Scale bars = 4 cm. PBB, progenitor biological bandage.
