# Supplementary material for: Retrospectives on Three Decades of Safe Clinical Experience with Allogeneic Dermal Progenitor Fibroblasts: High Versatility in Topical Cytotherapeutic Care
Source: Pharmaceutics. 2023 Jan 4;15(1):184. doi: 10.3390/pharmaceutics15010184 (PMC9866885; doi:10.3390/pharmaceutics15010184)
Supplement: Supplementary file 1 [file pharmaceutics-15-00184-s001.zip › Supplementary Tables.pdf]

Supplementary Tables

Table S1. Primary and secondary outcomes of the randomized clinical trials on allogeneic cutaneous progenitor cells listed in Table 1 of the manuscript. AE, adverse event; D, day; DSW, donor-site wound; M, month; PBB, progenitor biological bandages; SAE, serious adverse event.

Clinical Trial Name & References	Primary Outcomes of the Study (Results)	Secondary Outcomes of the Study
"The effects of acellular amniotic membrane loaded by cultured fetal fibroblast cells in split thickness skin wound healing";<br>Iran;<br>2013–2015;<br>IRCT201302218177N6	**1.** Frequency, type, severity of AE.<br>**2.** Pain: D 3, 5, 7, 9, 11, 15 after surgery, measured with a visual analogue scale.	**1.** Re-epithelialization rates: Examination and photography on D 3, 5, 7, 11, 13, 15, and 1 M after surgery; biopsy at D 7 after surgery. Sizes of treatment sites were 100±6 cm^2.<br>**2.** Infection: D 7 and D 15 after surgery, measured by examination and wound culture.<br>**3.** Scar: 3 M and 6 M after surgery, measured with a Vancouver scar scale.
"TWB-103 for adult patients with split-thickness skin graft donor site wounds";<br>Japan & Taiwan;<br>2017–2021;<br>NCT02737748	**1.** Incidence of treatment-related AE and SAE (including infections and bleeding, D 0–28).<br>**2.** The healing time from DSW creation to the first 100% re-epithelialization with confirmation for at least 10 days apart assessed by the investigator, D 42 or earlier.<br><br><u>Results</u>:<br>**1.** All of the subjects have achieved 100% re-epithelialization of their DSW within 2 weeks by receiving the study treatments for up to 7 days.<br>**2.** No study treatment-related AE or clinically significant changes of values in various measures of laboratory tests and vital signs were observed.	**1.** The healing time from DSW creation to the first 100% re-epithelialization with confirmation for at least 10 days apart, assessed by the first additional evaluator, D 42 or earlier.<br>**2.** The healing rates (complete wound closure) of patients at D 7, 10, and 14 after DSW creation. Complete wound closure is defined as skin 100% re-epithelialization without drainage or dressing requirements.<br>**3.** The healing percentage based on the healing area measured on D 7, 10, and 14, comparing to the original area measured on D 0 for all patients in the phase I and II trial.<br>**4.** The pain change from baseline to post-wound creation visits based on a short-form McGill pain questionnaire score, D 3, 7, 10, 14, 28, 42, and D 90/180/270/360 from D 28 or 42.<br>**5.** Incidence of AE and SAE, screening at D 360 from D 28 or 42. All incidence of AE and SAE will be analysed for all patients in the phase I and II trial.<br>**6.** Changes in post-treatment physical examination, vital signs, and general laboratory assessment compared to baseline, D 3, 7, 10, 14, 28, and 42.<br>**7.** Incidence of treatment-related AE and SAE (including infections and bleeding), screening at D 360 from D 28 or 42.

"Controlled comparison of a traditional dressing versus a biologic dressing composed of fetal fibroblasts and keratinocytes in association with a collagen matrix on skin donor sites"; France; 2018–2023; NCT03334656	**1.** The number of complete healings at D 8 judged by a physician observer. Healing is defined as 80% or more wound closure.	**1.** Concordance between the healing at D 8 (or D 11 and D 15) judged by a physician observer and by another physician using photographs, D 8 (or D 11 and D 15 if the healing is not completed). Healing is defined as 80% or more wound closure.<br>**2.** Wound healing's rapidity of CICAFAST versus conventional treatment (Jelonet) in the treatment of the donor site, on D 8 and D 11 (if the healing is not completed), and D 15 (if the healing is not completed) judged by a physician observer.<br>**3.** Tolerance of CICAFAST versus the conventional treatment (Jelonet) after 6 M.<br>**4.** Pain of the wound healing with CICAFAST versus conventional treatment (Jelonet), D 8 and D 11 (if the healing is not completed), and D 15 (if the healing is not completed).<br>**5.** Quality of the wound healing with CICAFAST versus conventional treatment (Jelonet) after 6 M. Healing is defined as 80% or more wound closure.<br>**6.** Quality of the wound healing with CICAFAST versus conventional treatment (Jelonet) for the patients who will have confocal microscopy after 3 M.
"Evaluation of the safety and effectiveness of progenitor biological bandages in burn care"; Switzerland; 2022–2032; NCT05339490	**1.** Wound re-epithelialization assessment at D 10. 95% skin re-epithelialization (yes or no) will be assessed 10 D after the start of treatment and creation of the DSW.	**1.** Short-term efficacy of treatment at D 5, 10, and 15 of treatment. Re-epithelialization in % compared to D 1 of treatment.<br>**2.** Long-term skin quality - Scar appearance (Vancouver Scar Scale), from 1 M to 5 years post skin closure.<br>**3.** Long-term skin quality, scar colour, from 1 M to 5 years post skin closure.<br>**4.** Long-term skin quality, elastography, from 1 M to 5 years post skin closure.<br><br><u>Other Outcome Measures</u>:<br>**1.** Wound infections. Through study treatment, an average of 15 D for each treated wound.<br>**2.** AE. Through study completion, an average of 5 years.

Table S2. Summarized overview of details on clinical protocols and clinical trials approved and conducted between 1999 and 2022 around the cytotherapeutic uses of PBBs in the CHUV (Lausanne, Switzerland). CER, Vaud cantonal ethics commission for research on human subjects; CHUV, centre hospitalier universitaire vaudois; NA, non-applicable; PBB, progenitor biological bandages.

Year	Clinical Protocol or Study Reference	Clinical Protocol or Study Title	Principal Investigators
1999	F-33/99 CER	"Efficacy of biological bandages with fetal cells on pediatric burn patients and on adult chronic cutaneous wounds"	Dr. Judith Hohlfeld
2000	F-119/00 CER	"Treatment of extensive burns with cutaneous allografts" [1]	Dr. Judith Hohlfeld
2007/2013	62/07 CER	"Development of fetal cell banks for tissue engineering"	Prof. Lee Ann Laurent-Applegate
2012	199/12 CER	"Study of the efficacy of biological bandages on the healing of standardized wounds"	Prof. Wassim Raffoul
2017	2017-01796 BASEC	"Retrospective study on the treatment of burned children in the CHUV and the use of biological bandages"	Dr. Anthony de Buys Roessingh
2022	*BRU_PBB*; 2020-01873 BASEC; 2020TpP1010 Swissmedic; NCT05339490	"Evaluation of the safety and effectiveness of progenitor biological bandages in burn care"	Dr. Anthony de Buys Roessingh

[1] Amended in 2001 to "Treatment of extensive burns and chronic wounds with cutaneous allografts".

Table S3. Overview of the components of each of the considered progenitor cytotherapies, along with therapeutic indications, cell dosing considerations, and modalities of product reconstitution for clinical administration. No placebo formulations of the PBB, PBI, or ePBB were used in the documented clinical work. API, active pharmaceutical ingredient; ePBB, equine progenitor biological bandage; FPC, fibroblastic progenitor cells; h, hours; PBB, progenitor biological bandage; PBI, progenitor biological bandage yielding γ-irradiated cells.

Type of Topical Cytotherapy	Treatment Description	Therapeutic Indications	Product Dose & Reconstitution Method
**PBB**	Viable allogeneic human dermal progenitor fibroblasts in cryopreservation solution (API) + equine collagen matrix (functional scaffold)	Primary burn wounds, donor-site wounds, chronic limb ulcers	Initiation of APIs from cryopreservation and seeding at 4.5×10^3 cells/cm^2 on the collagen scaffold; incubation for 18–24 h at 37 °C
**PBI**	Viable and growth-arrested (i.e., 200 Gy irradiation) allogeneic human dermal progenitor fibroblasts in cryopreservation solution (API) + equine collagen matrix (functional scaffold)	Primary burn wounds, donor-site wounds	Initiation of APIs from cryopreservation and seeding at 4.5×10^3 cells/cm^2 on the collagen scaffold; incubation for 18–24 h at 37 °C
**ePBB**	Viable allogeneic equine progenitor fibroblasts in cryopreservation solution (API) + equine collagen matrix (functional scaffold)	Primary burn wounds, traumatic wounds	Initiation of APIs from cryopreservation and seeding at 5×10^3 cells/cm^2 on the collagen scaffold; incubation for > 24 h at 37 °C
